# The Prion-like protein Shadoo is involved in mouse embryonic and mammary development and differentiation

**DOI:** 10.1038/s41598-020-63805-y

**Published:** 2020-04-21

**Authors:** Bruno Passet, Johan Castille, Samira Makhzami, Sandrine Truchet, Anne Vaiman, Sandrine Floriot, Katayoun Moazami-Goudarzi, Marthe Vilotte, Anne-Laure Gaillard, Louise Helary, Maud Bertaud, Olivier Andréoletti, Daniel Vaiman, Pierre Calvel, Nathalie Daniel-Carlier, Mohammed Moudjou, Christian Beauvallet, Mohamed Benharouga, Denis Laloé, Sophie Mouillet-Richard, Amandine Duchesne, Vincent Béringue, Jean-Luc Vilotte

**Affiliations:** 1 Université Paris-Saclay, INRAE, AgroParisTech, UMR1313-GABI, 78350 Jouy-en-Josas, France; 2 Université Paris-Saclay, INRAE, UVSQ, VIM, 78350 Jouy-en-Josas, France; 3 ISC3T, UMR1225, INRAE, ENVT, 31076 Toulouse, Cedex France; 4 Institut Cochin, U1016, INSERM, UMR 8504 CNRS, Université de Paris, Paris, France; 5 LCBM-UMR5249, DRF-BIG, CEA-Grenoble, 38054, Grenoble, Cedex 09 France; 6 Centre de Recherche des Cordeliers, INSERM, Sorbonne Université, Université de Paris, F-75006 Paris, France

**Keywords:** Developmental biology, Development

## Abstract

Shadoo belongs to the prion protein family, an evolutionary conserved and extensively studied family due to the implication of PrP in Transmissible Spongiform Encephalopathies. However, the biological function of these genes remains poorly understood. While *Sprn*-knockdown experiments suggested an involvement of Shadoo during mouse embryonic development, *Sprn*-knockout experiments in 129Pas/C57BL/6J or 129Pas/FVB/NCr mice did not confirm it. In the present study, we analyzed the impact of *Sprn* gene invalidation in a pure FVB/NJ genetic background, using a zinc finger nuclease approach. The in-depth analysis of the derived knockout transgenic mice revealed a significant increase in embryonic lethality at early post-implantation stages, a growth retardation of young *Sprn*-knockout pups fed by wild type mice and a lactation defect of *Sprn*-knockout females. Histological and transcriptional analyses of knockout E7.5 embryos, E14.5 placentas and G7.5 mammary glands revealed specific roles of the Shadoo protein in mouse early embryogenesis, tissue development and differentiation with a potential antagonist action between PrP and Shadoo. This study thus highlights the entanglement between the proteins of the prion family.

## Introduction

The prion protein, encoded by *Prnp*, is extensively studied due to its implication in Transmissible Spongiform Encephalopathies (TSE). Indeed, the misfolded, partially protease-resistant, PrP conformer is the main, if not the sole, component of prions, the infectious agent responsible for TSE in animals and humans^1^. The evolutionary conservation of PrP suggested its involvement in essential cellular processes^2^. *Prnp* is ubiquitously expressed throughout development and adult life, with a higher expression level in the central nervous system. Surprisingly, naturally occurring or generated mammals or fish devoid of PrP develop normally^3–6^, suggesting that (an)other host-encoded protein(s) induces() compensatory mechanism(s).

Two other members of the mammalian prion family were described, Shadoo, encoded by *Sprn* and homologous to the N-terminal part of PrP, and Doppel, encoded by *Prnd* and homologous to the C-terminal part of PrP^7^. In the adult, Doppel is mainly expressed in male gonads and *Prnd* or *Prnd*/*Prnp* knockout male mice are sterile^8^.

Shadoo shares with PrP some spatial regulation and properties^9^. The knockdown of *Sprn* in 129Pas/FVB/NCr *Prnp*-knockout embryos induces a lethal phenotype at early embryonic stages (~E10)^10,11^, originating from a trophectoderm-derived compartment developmental failure^11^. However, *Sprn* or *Sprn*/*Prnp* co-ablation in 129Pas/C57BL/6J or 129Pas/FVB/NCr mice did not reveal embryonic developmental abnormality^12^. These divergent observations, sometimes attributed to genetic compensation in mutated animals^13^, questioned the involvement of Shadoo during embryogenesis.

In this study, we report the establishment, through injection of a Zinc Finger Nuclease (ZFN), of FVB/NJ *Sprn*-knockout (*Sprn*^*0/0*^) mice. Reproductive performances and transcriptomic analyses of these mice demonstrate that Shadoo is involved in embryonic development and more generally in the development and differentiation of several tissues. Furthermore, our data suggest antagonist actions of PrP and Shadoo, at least during embryonic stages.

## Results

### Generation of Sprn^0/0^ mice

**Figure 1:**
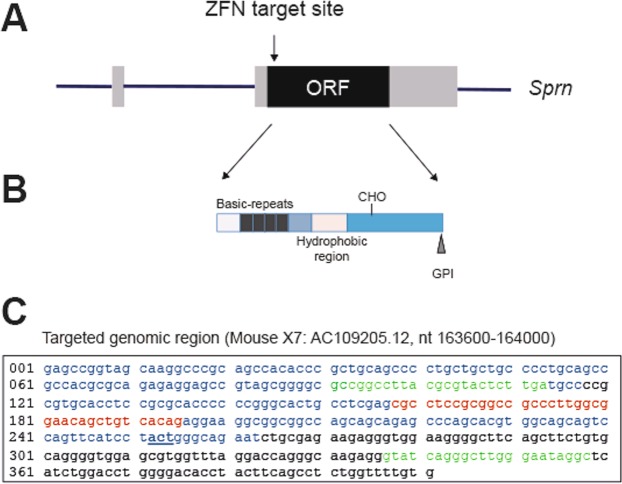
Schematic representation of the *Sprn* gene and location of the ZFN target site. (**A**) Schematic representation of the *Sprn* gene. Bar: flanking and intronic sequences. Grey boxes: exonic untranslated regions. Black box: Open Reading Frame (ORF) sequence. Location of the ZFN target site is indicated on the top. The scheme is not at scale. (**B**) Representation of the Shadoo protein. (**C**) Location of the ZFN target site. Blue sequence: partial *Sprn* exon 2. Red sequence: ZFN target site. Green sequences: oligonucleotides used for genotyping analysis. Bold, underlined nucleotides: start of *Sprn* ORF (reverse orientation).

The ZFN target site is located at the start of the *Sprn* ORF (Fig. 1). ZFN mRNA was injected into 1 cell-fertilized FVB/NJ mouse eggs that were transferred into pseudo-pregnant mice. Tail-DNA analysis of the 29 resulting pups by PCR amplification of a ~400 bp genomic region surrounding the ZFN-target site and sequencing analysis allowed identifying 9 transgenic founder mice, carrying small deletions ranging from 1 to 13 bp. All founder mice were heterozygous for the mutated allele.

**Figure 2:**
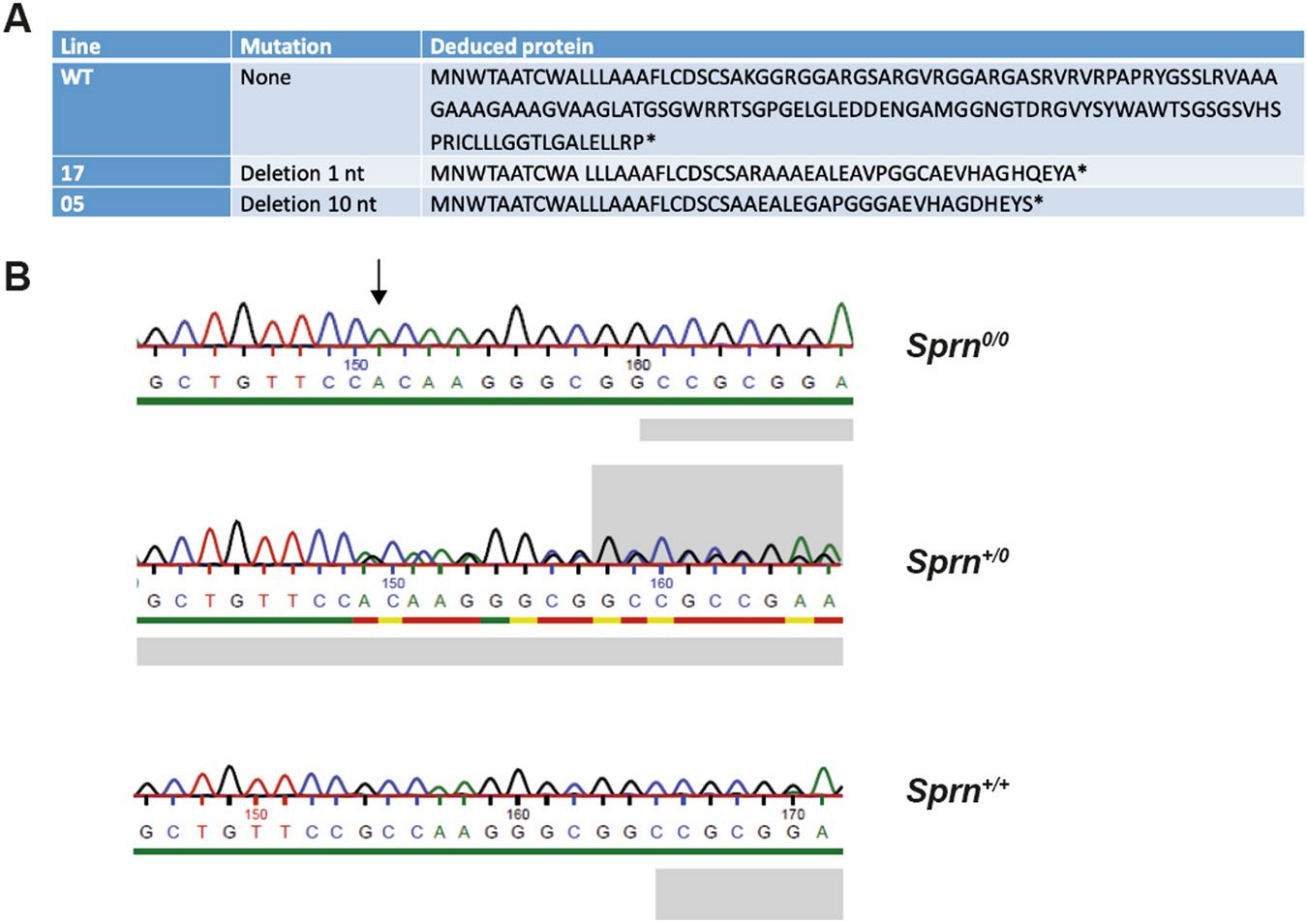
Sequence analysis of mutant mice. (**A**) Sequence of Shadoo and protein sequences of mutant *Sprn* genes from lines 17 and 05. *end of the protein sequence. (**B**) Sequence analysis of the *Sprn* genotypes in line 17 following PCR amplification of the mutated region.

Offspring from two founders (17 and 05) were used to generate *Sprn*^*0/0*^ mice. These founders were chosen as they transmitted a mutated allele (deletion of 1 nucleotide associated with the mutation of an adjacent nucleotide and deletion of 10 nucleotides, respectively), inducing a frameshift leading to an early premature stop codon. Translation of the corresponding mRNA is predicted to produce truncated nonsense proteins (Fig. 2) lacking all Shadoo domains (Fig. 1).

Intercrosses of *Sprn*^*0/+*^ mice produced *Sprn*^*0/0*^ mice (Table 1). Knockout mice appear normal and fertile, as previously observed^12^. However, the percentage of *Sprn*^*0/0*^ mice was significantly lower than expected (Table 1), suggesting embryonic lethality. Intercrosses of *Sprn*^*0/0*^ mice led to litters with an average size of 6.44 (174 pups/27 litters; 54/8 for line 05, 120/19 for line 17). This litter size was significantly smaller compared to FVB/NJ mice (8.6 pups/litter; 215/25, t-test p < 0.05). To assess the fertility of *Sprn*^*0/0*^ males and females, crosses between *Sprn*^*0/0*^ and WT mice were studied. The average litter sizes were of 9.7 (88/9) and 9.8 (59/6) for *Sprn*^*0/0*^ males and females, respectively. These results were similar to those of FVB/NJ mice, suggesting that *Sprn* knockout is not associated with sub-fertile sex-related phenotype or inability of *Sprn*^*0/0*^ females to sustain normal gestations. Of note, such a decrease in litter size was reported for *Prnp*-overexpressing mice^14^. As both *Sprn*^*0/0*^ lines behaved similarly (average litter sizes, transmission rate), line 17 was chosen for further detailed analyses. It is referred to as *Sprn*^*0/0*^ thereafter.

**Table 1:** Analysis of pup genotypes following crossing of heterozygous mice.

*Parents: Sprn*^+*/0*^ × *Sprn*^+*/0*^	Observed pups’ genotypes	Expected (Mendelian transmission)
*Sprn*^+/+^	47	39.5
*Sprn*^+*/0*^	85	79
*Sprn*^*0/0*^	26*	39.5

*t-test P < 0.05.

*Sprn* expression analysis was investigated in *Sprn*^*0/0*^ mouse brains. *Sprn*-transcript levels seemed unaffected, as judged by semi-quantitative RT-PCR experiments (data not shown). This observation was expected since the ORF is located within the last exon of the gene and the mutated transcript should thus escape nonsense mediated decay^15^. Microarray transcriptomic analysis of other tissues led to similar observations as *Sprn* was not detected as differentially expressed between *Sprn*^*0/0*^ and FVB/NJ mice (see below). The entire ORF was reverse-transcribed from brain RNAs of WT and *Sprn*^*0/0*^ mice. No alternative splicing was detected and *Sprn* ORF from *Sprn*^*0/0*^ mouse mRNAs carried the expected nonsense mutation (Fig. S1A). Of note is the lack of downstream in frame ATG codon close to the nonsense mutation, avoiding translation re-initiation (Fig. 2). Brain western blots analyses did not allow detecting Shadoo in WT or *Sprn*^*0/0*^ brains, despite using several commercially (Abgent # AP4754b, PA5 24688, ab175070) or laboratory homemade (1909 S0, S4 et S5, J74 et J740 from CEA, France and 06sh1 from University Alberta, Canada) antibodies and detection techniques (ECL, IF, data not shown). Nevertheless, RNA investigations clearly demonstrate the knockout genetic status of these mice.

### Increased lethality of Sprn^0/0^ embryos

We next investigated whether the reduced litter size in *Sprn*^*0/0*^ mice was associated with increased embryonic lethality. Pregnant females from *Sprn*^*0/0*^ × *Sprn*^*0/0*^ or WT × WT crossings were analyzed at G7.5 and G14.5. At G7.5, no resorption was observed in FVB/NJ mice (0/76 implantation sites (IS)). In contrast, a statistically significant 12.7% resorption rate was detected in *Sprn*^*0/0*^ females (7/55 IS, p < 0.05). Empty decidua suggested that resorptions occurred early after implantation. At G14.5, this significant difference remained with a resorption rate of 19.7% in *Sprn*^*0/0*^ mice (14/71 IS) compared to 6.2% in WT mice (10/162 IS). Thus, the reduced litter size observed in *Sprn*^*0/0*^ × *Sprn*^*0/0*^ crossings results from an increased fragility of the embryos at early post-implantation, a key period of mammalian fetal development.

### Transcriptomic analysis of E7.5 Sprn^0/**0**^ embryos

To get deeper insight into the biological consequences of *Sprn* knockout in embryonic development, comparative microarray transcriptomic analysis of WT and *Sprn*^*0/0*^ embryos was performed at E7.5. Following raw data normalization, differentially expressed genes (DEG) with a p-value < 0.05 and a fold change (fc) <−2 or >2 were analyzed. Overall, 147 DEG were identified, 86 upregulated and 61 downregulated, of which 29 were small nucleolar RNAs (Table S1). These small RNA genes regulate cell proliferation, migration and invasion by inducing Epithelial-Mesenchymal transition (EMT)^16,17^. Notably, a large set of small nucleolar RNAs with a conserved H/ACA box (snoRA) were downregulated. Such downregulation was found to be associated with cellular apoptosis^17^. Further, gene set enrichment analysis (GSEA) identified downregulation of genes involved in dorsal spinal cord development (enrichment score (ESc) 3.37, false discovery rate (FDR) 0.006), mitochondrial translational termination and elongation (ESc 2.7, FDR 3.08 × 10^−5^), constituent of ribosomes (ESc 3.47, FDR 4.5 × 10^−17^) and proteasome core complex (ESc 2.6, FDR 4.2 × 10^−5^). Identification of these biological pathways revealed that *Sprn*^*0/0*^ E7.5 embryos suffer from developmental defects.

Ingenuity® Pathway Analysis (IPA®) (http://www.ingenuity.com/) of the 147 DEG identified 4 significant networks (score > 22); developmental disorder, cell-to-cell signaling and interaction, immunological and/or hematological disease, organism survival and cell growth and proliferation (Table 2). It suggested that Shadoo deficiency alters cell proliferation and differentiation resulting in abnormal early development. Potential related master regulator genes were identified in further analyses of the DEG, but at reduced fc (note S1).

**Table 2:** Identified networks with differentially expressed genes in E7.5 embryos.

Network	Differentially expressed genes in E7.5 embryos
Developmental Disorder	**AOC1**,**C3**, **C5AR2**, **CASP8**, **CAT**, **CAV1**, **CD68**, **Cyb5r3**, **CYBB**, **DCN**, **DES**, **EHD2**, **FABP4**, **GLP1R**, **GZMH**, *HSPA1A/HSPA1B*, **ITGA2**, **LY96**, *mir-15*, **Ms4a4b**, **NFYA**, **PGF**, **SLC25A12**, **SLC6A4**
Cell-to-cell signaling and interaction	**ANXA11**, **C1S**, **DAG1**, **FADS3**, **IRAK3**, **LAPTM5**, **LY96**, **MFSD2A**,*mir-302*, **OSMR**, **RNASE4**, **SLC38A3**, **Srgn**, **TNS4**, **ZFAND2A**
Immunological and/or hematological Disease	**AGPAT2**, **AMPD3**, *ATP6V1G1*, **C3**, **Gstm3**, **IFI44**, *LSM3*, *MALAT1*, *mir-17*, *mir-19*, **MUC13**,*Neat1*, **Prl2c2**, *RPL14*, *Rpph1*, **SIKE1**
Organismal survival	**ALG12**, **COQ5**, **EHD3, Erv3**, **FERMT1**, **FSTL3**, **GPX3**, **GREM2**, *Hspa1b*, *mir-467*, **MPEG1**, **PAPSS2**, **Serpinb9f**, **SLCO2A1**
Cellular Growth and Proliferation	**BIN2**, **DHRS9**, **Ear2**,*mir-17*, **NFYA**, *PEG10*, *RBM3*, **SCUBE2**, **SDC3**, *SNORA74A*, *Snord118*, **Speer4a**, **TC2N**, **Tpsab1**

Bold-faced genes: positively differentially expressed genes in *Sprn0/0* embryos. Underlined genes: negatively differentially expressed genes in *Sprn0/0* embryos. Yellow colored genes: genes also found differentially expressed in Prnp0/0 E7.5 embryos^11^. Of note, these genes were all differentially expressed in an opposite way between *Sprn0/0* and *Prnp0/0* embryos.

Collectively, these transcriptomic analyses suggest that *Sprn* knockout induces alteration of EMT pathways, impacting cell fate determination and inducing embryonic lethality soon after the implantation.

### Potential opposite transcriptomic alterations between Sprn and Prnp knockout E7.5 embryos

Some pathways identified above are reminiscent of those described for PrP modulation of EMT^18,19^. PrP regulates embryonic stem cell pluripotency and differentiation, and Shadoo and PrP share biological properties^9^ although recent experiments suggest that it could depend of the model system used^20,21^. As we previously performed transcriptomic analysis of E7.5 *Prnp*^*0/0*^ embryos^11^, we searched for potential shared DEG between *Prnp*^*0/0*^ and *Sprn*^*0/0*^ embryos. Two master regulators of pluripotency were differentially expressed in E7.5 *Prnp*^*0/0*^ embryos, Oct4 and TGFβr2. Oct4 was overexpressed and TGFβr2 downregulated in *Prnp*^*0/0*^ embryos^11^, i.e. oppositely to what is observed in *Sprn*^*0/0*^ (note S1). Furthermore, out of the 147 DEG observed in *Sprn*^*0/0*^ embryos, 12 were also differentially expressed in *Prnp*^*0/0*^ embryos^11^ but in an opposite sense, those indicated in Table 2 alongside *Hspb7* and *Igsf11* (Table S1). Importantly, *Prnp* was not differentially expressed in *Sprn*^*0/0*^ embryo, excluding this observation be an indirect consequence of *Prnp* overexpression.

Altogether, these observations suggest that Shadoo and PrP exert antagonist functions in early embryonic development. It fits in with the above-mentioned observation that *Sprn*^*0/0*^ and *Prnp*-overexpression^14^ both induces smaller average litter sizes. However, these opposite differential gene expressions involved a limited number of genes and small variation levels, and we cannot formally exclude that this observation made across different experiments, might be coincidental.

### Comparative analyses of Sprn^0/0^ pups’ growth curves and of Sprn^0/0^ placentas

**Figure 3:**
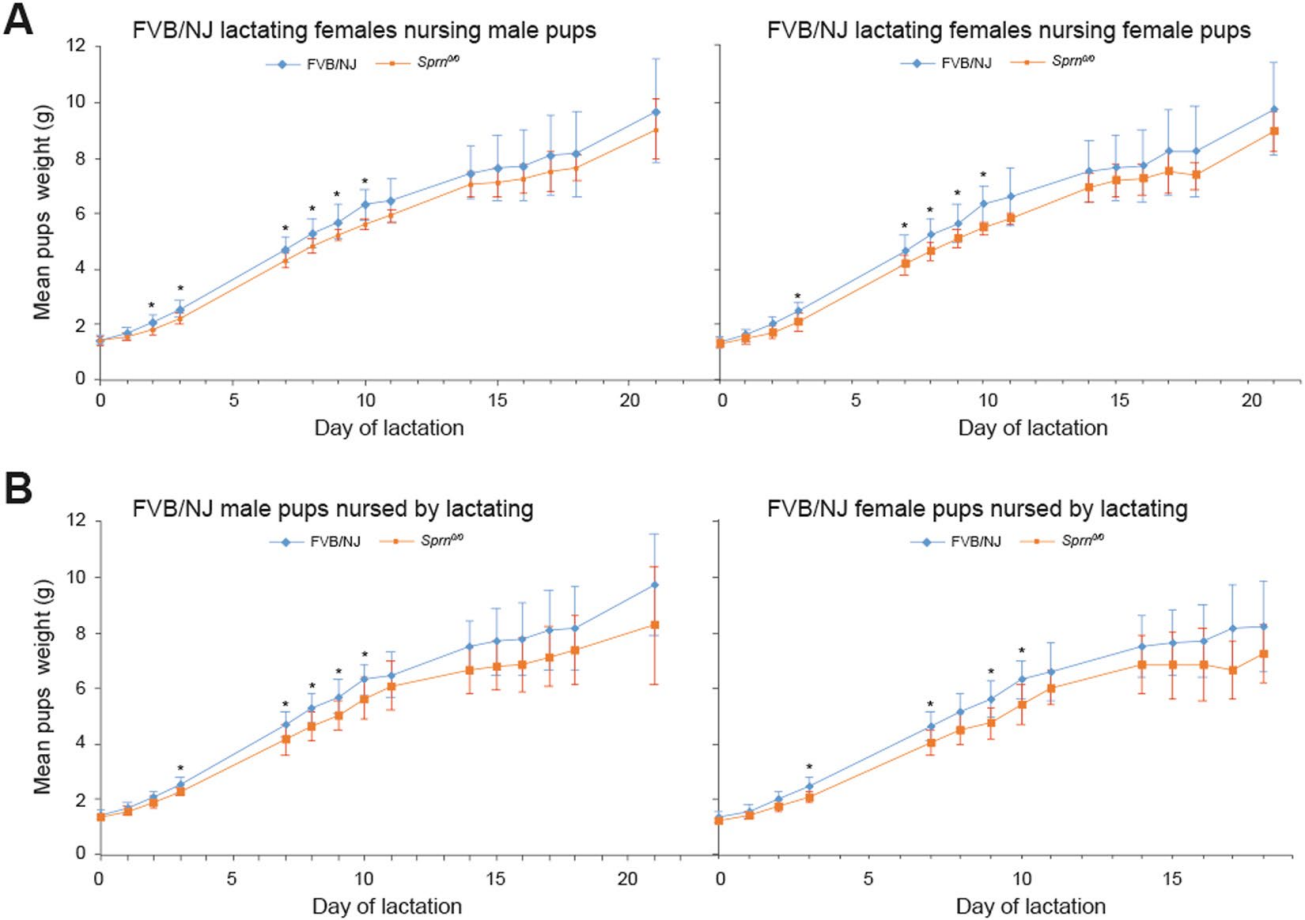
Analysis of pup’s growth curves. (**A**) Analysis of potential growth differences according to the genotype and sex of the pups. Male or females pups from FVB/NJ (blue) or *Sprn*^*0/0*^ (orange) genotype were fed by FVB/NJ females. Statistically different mean weights between pups according to their genotypes (t test p < 0.05) are indicated by *. At least 6 pups of each genotypes for both sexes were weighted per stage (see Material and Method section) (**B**) Analysis of potential lactating differences according to the genotype of the females. FVB/NJ male or female pups were fed by either FVB/NJ (blue) or *Sprn*^*0/0*^ (orange) females. Statistically different mean weights between pups fed by the two types of lactating female (t test p < 0.05) are indicated by *. At least 6 pups of each genotypes for both sexes were weighted per stage (see Material and Methods).

To assess whether pups body weight is affected in *Sprn*^*0/0*^ mice, as previously reported^12^, we compared the growth curves of WT and *Sprn*^0/0^ pups fed by WT lactating females. The body weight gain was lower for *Sprn*^*0/0*^ versus WT pups (statistically significance between L3-L10, Fig. 3A). At variance with the previous data^12^, there was no difference according to the pup sex. Abnormal growth could have various origins, one being placental dysfunction as in *Prnp*^*0/0*^ mice^14^. Placental dysfunction would explain that at birth, the weight of the *Sprn*^*0/0*^ pups were significantly lighter (1.34 +/− 0.14 g for *Sprn*^*0/0*^ pups (n = 45) versus 1.4 +/− 0.16 g for WT pups (n = 97), t-test p < 0.05), suggesting intra-uterine growth retardation. We thus analyzed whether *Sprn* knockout affects placental efficiency. Fetuses and placental tissues from E14.5 WT and *Sprn*^*0/0*^ mice were weighted. No significant difference was observed between the two genotypes (Table 3). Histological examination of E14.5 and E17.5 placentas did not reveal structural or vascularization modification (data not shown). The relative proportions of the labyrinth layer, spongiotrophoblast and decidua zones were unaffected.

**Table 3:** Analysis of placenta and fetal weights.

Mouse genotype	FVB/NJ	*Sprn*^*0/0*^
Placenta Weight E14,5 (average +/− SEM)	0.064 ± 0.008	0.065 ± 0.012
Fetal Weight E14,5 (average +/− SEM)	0.236 ± 0.029	0.230 ± 0.034
Placental efficiency	3.754 ± 0.526	3.585 ± 0.580
Number of analyzed Pups	66	63
Number of analyzed Litters	7	11

We next performed comparative transcriptomic analysis on E14.5 WT and *Sprn*^*0/0*^ placentas. Following raw data normalization, DEG with a p-value < 0.05 and a fc < −2 or > 2 were selected. 133 genes were identified, of which 17 were downregulated (Table S2), including 12 small nuclear RNAs. Seven DEG were common between placentas and embryos, all of them behaved similarly between the two tissues; 4 small nuclear RNAs alongside *Glp1r*, *LY96* and *SLCO4C1* genes. This small number of shared DEG was expected due to transcriptomic divergences between organs besides early stages of development^22^. Validation of the transcriptional regulation of five genes, TBPA, Gli3, GC, PSG23, SerpinA1 (α1—antitrypsin), was tested by RT-qPCR. All results were concordant (Table S3). Furthermore, immunoblot analyses were performed to examine the expression levels of two proteins, SerpinA1 and Fibrinogen. The results confirmed the tendency observed at the transcriptomic levels, with on average a higher expression level of the two proteins in the knockout animals, which did not reach statistical significance (Fig. S2).

IPA® of the DEG identified two major associated networks (score > 35); lipid metabolism, molecular transport, small molecule biochemistry and tissue development, cellular movement (Table 4). Global gene activation within these networks led to the overexpression of various protein families, including apo-lipoproteins and fibrinogens. Both gene families exert protective effects against stresses, supporting placentation and embryonic development^23,24^. Potential related master regulator genes were identified in further analyses of DEG, but at reduced fc (note S2).

**Table 4:** Identified networks with differentially expressed genes in E14.5 placentas.

Network	Differentially expressed genes in E14.5 placentas
Lipid metabolism, Molecular transport, Small molecule biochemistry	**Acnat1/Acnat2**, **ADH1C, AHSG, Akr1b7, AKR1C3**, **ALB**, **ALDOB, AMBP**, **APOA1**, **APOA2**, **APOA4**, **APOB**, **APOC2**, **Apoc3**, **APOM**, **C6, C9, CDHR2, CFI, Clec2e/Clec2h, CPN1, CUBN**, **F2**, **FCGRT**, **FGA**, **FGB**, **FGG**, **GC**, **GLP1R**, **GSTK1, Hamp/Hamp2**, **KNG1**, **LRP2**, **PCBD1, RBP4**, **SCGB1A1**, **SERPINA10, SERPIND1**, **SLC22A2, SLC5A1, SLCO1B3, TF**, **TTR**, **UGT2B10, VTN**
Tissue development, Cellular movement	**AGT**, **AHSG**, **Akr1b7**, **APOH**, **BST1, C10orf10**, *CD300LD*, **CIDEB**, **CMBL**, **CPS1**, **CYP21A2**, **DAD1**, **FGG, Fxyd2**, **GIPC2, HABP2**, **HBB**, **HGD, HSD3B2**, **KLK3, LY96**, **NDUFA3**, **PDZK1IP1, PLG**, **PLGRKT**, *Psg18 (includes others)*, **SERPINA1**, **SLC27A2**, **SLC2A2**, **SLC3A1**, **STRA13, Tceb2, VDR**

Bold-faced genes: positively differentially expressed genes in *Sprn0/0* placentas. Italized genes: negatively differentially expressed genes in *Sprn0/0* placentas.

Altogether, these data suggest that at E14.5, *Sprn*^*0/0*^ placentas underwent an adaptive biological process, involving LXR/RXR and FXR/RXR activation highlighted by the apo-lipoproteins and serpinA1 upregulations, to compensate earlier developmental dysfunctions.

### Sprn-invalidation affects mouse lactation

Since *Sprn* appears to be involved in embryonic EMT and in cellular proliferation and tissue development and since PrP^C^ is involved in the self-renewal of mammary adult stem cells^25^, we assessed whether *Sprn* knockout affects lactation. We compared the growth curves of WT pups fed by WT or *Sprn*^*0/0*^ lactating females. Their weight gain was significantly lower when fed by *Sprn*^*0/0*^ females between L2 and L10 (Fig. 3B). No significant difference was observed between WT and *Sprn*^*0/0*^ milk protein concentrations at L7.5 (Table S4). Consistently, SDS-PAGE and western analyses did not reveal modification of the milk protein profiles (Fig. S3). Iron, zinc and copper concentrations were also similar between *Sprn*^*0/0*^ and WT milks (data not shown). These data thus suggest that *Sprn* invalidation affects mouse lactation.

### Sprn-invalidation affects milk fat globules volumes and morphology

**Figure 4:**
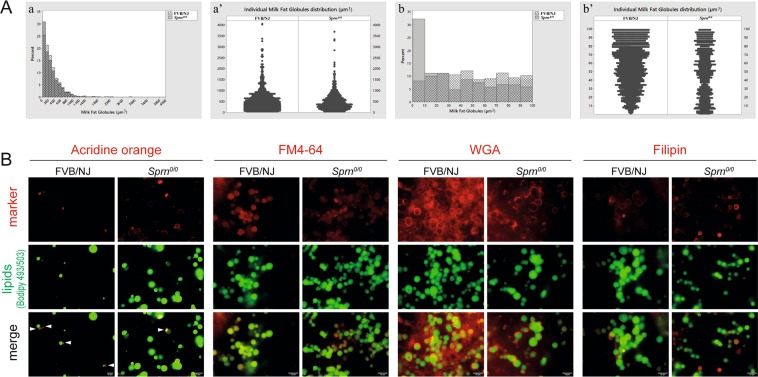
Effects of *Sprn* invalidation on Milk fat globules volume and morphology. (**A**) (a,a’) Comparative representations of the milk fat globule volumes distribution between FVB/NJ and *Sprn*^*0/0*^ milks. At least 3 individual milks were analyzed per genotype, as described in the Material and Methods section. (**A**) (b,b’) Comparative representations of 0 to 100 µm^3^ milk fat globule distribution between FVB/NJ and *Sprn*^*0/0*^ milks. At least 3 individual milks were analyzed per genotype, as described in the Material and Methods section. (**B**) Immunofluorescence imaging of MFGs from FVB/NJ or *Sprn*^*0/0*^ mice at day 10 of lactation. MFGs were co-stained for for neutral lipids (green) with Bodipy 493/503 and (red) for either cytoplasmic crescents (arrowheads) with acridine orange, membranes with FM4–64, glycoproteins with wheat germ agglutinin (WGA) or free cholesterol with filipin. Bar, 10 µm.

Analysis of milk fat globules (MFGs) revealed a significant reduction of the medians volume of *Sprn*^*0/0*^ MFGs (n = 3166) compared to that of FVB/NJ MGFs (n = 5643) (Fig. 4A, kruskal-Wallis chi-squared = 22.114, p-value = 2.57 10^−06^). Analysis of the size distribution of the MFGs showed that the majority of MFGs were smaller in *Sprn*^*0/0*^ milk with a significant increase (up to 30% of total MFGs) of very small MFGs (size between 0 and 100 µm^3^, Fig. 4Aa). Intriguingly, the detailed analysis of this size range (Fig. 4A,b,b’) showed that more than 30% of the very small MFGs in *Sprn*^*0/0*^ milk were <10 µm^3^ (Fig. 4Ab). Moreover, while size distribution of MFGs from FVB/NJ mice between 0 and 100 µm^3^ was in favor of the largest ones, those from *Sprn*^*0/0*^ mice were evenly distributed (Fig. 4Ab’). To what extend this difference in MGF size distribution affects milk digestibility and pups’ growth remain unknown. Nevertheless, these observations suggest that the growth of intracellular lipid droplets (CLDs), potentially linked to their apical transport and secretory mechanisms, could be altered in the absence of Shadoo. We thus investigated whether Shadoo absence affects MFGs morphological properties. MFGs were co-labeled with Bodipy 493/503 for neutral lipids and either acridine orange for cytoplasmic crescents, FM4-64 for membranes, wheat germ agglutinin for glycoproteins and filipin for free cholesterol, before analysis by immunofluorescence microscopy (Fig. 4B). While MFGs from *Sprn*^*0/0*^ and FVB/NJ mice exhibited similar proportions of cytoplasmic crescents, those from *Sprn*^*0/0*^ mice were rather labeled at their periphery, suggesting a more acidic environment (Fig. 4B, AO). MFGs membranous morphology and surface glycoproteins distribution was similar in both genotypes (Fig. 4B, FM4-64; WGA). However, MFGs from *Sprn*^*0/0*^ mice appeared enriched in free cholesterol, a hallmark of membrane microdomains called rafts (Fig. 4B, filipin). Collectively, these results point to subtle modifications of MFGs membranes from *Sprn*^*0/0*^ mice, which may impact their size distribution^26,27^.

### Sprn invalidation affects mouse mammary epithelial tissue development

**Figure 5:**
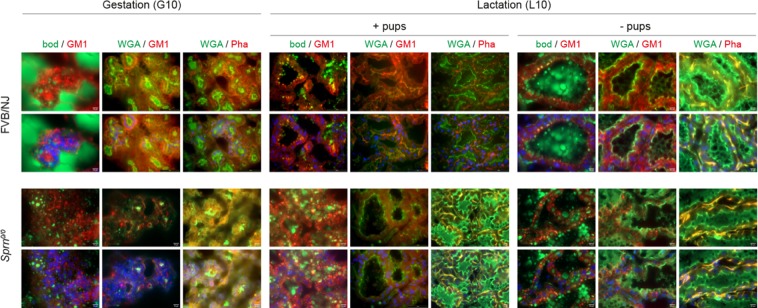
Mammary gland morphology and mammary epithelial cell architecture. Confocal microscopy imaging of mammary gland sections from FVB/NJ and *Sprn*^*0/0*^ mice at mid-gestation (G10) and full lactation (L10, in the presence or absence (±pups) co-stained for neutral lipids (Bodipy 493/503 (bod), green), rafts microdomains (cholera toxin B subunit (GM1), red), glycoproteins (wheat germ agglutinin (WGA), green), and/or actin (Phalloïdin (Pha), red). Nuclei were counterstained with nuclear marker 4′,6-diamidino-2-phenylindole (DAPI, blue). The Asterisks indicate the lumen. Bar, 10 µm.

As *Sprn*^*0/0*^ MGFs morphological properties were altered, we investigated whether differences could be observed at the MG level. Confocal imaging on tissue sections were performed at three MG developmental stages: mid-gestation (G10) and full lactation (L10) in the presence or absence of pups. Key features such as lipid droplets morphology, apical polarity and organization of the myoepithelial cells were analyzed (Fig. 5). At these stages, the lipid droplets, while correctly associated with the raft specific marker GM1 ganglioside, were generally smaller in *Sprn*^*0/0*^ mammary epithelial cells with a more disperse distribution. The myoepithelial cells morphology and general organization around forming or active alveoli was markedly disturbed in *Sprn*^*0/0*^ mice, as assessed by glycoproteins (WGA) and actin (PHA) staining. These data thus suggest that the lack of shadoo disturbs the establishment of the apico-basal polarity of mammary epithelial cells and alveoli formation.

### Sprn-invalidation affects mouse mammary proliferation

**Figure 6:**
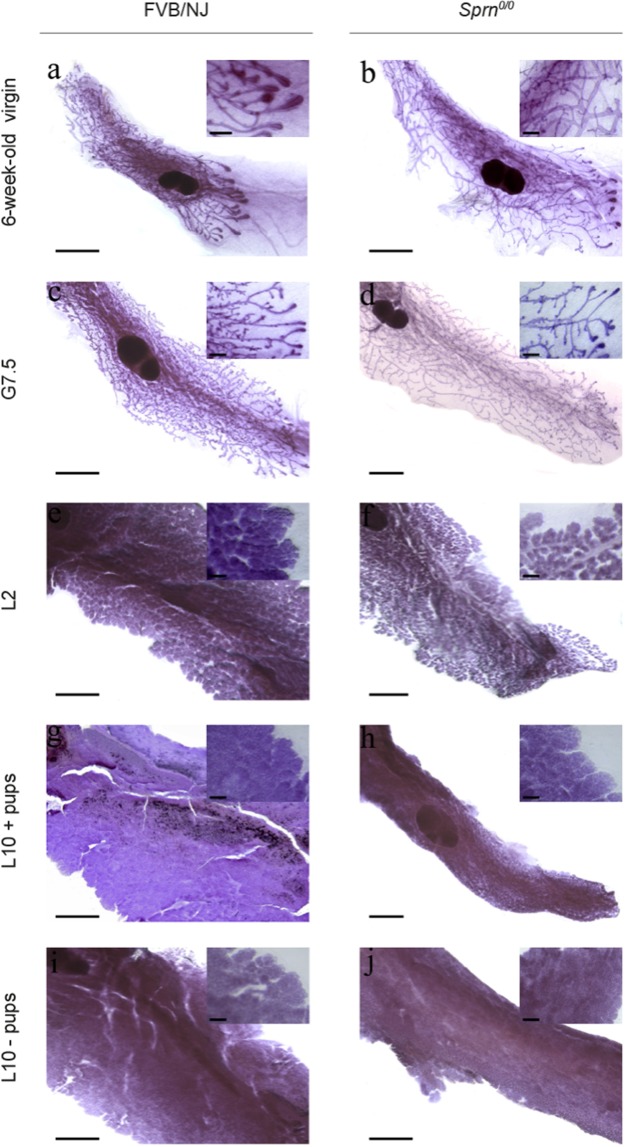
Mammary gland development analysis. Representative whole mount analysis of mammary gland structure from WT (FVB/NJ; **a,c,e,g,i**) or mutant (*Sprn*^*0/0*^, **b,d,f,h,j**) mice. It highlights defects in duct tree formation in 6-week-old virgin, in duct side-branching during early gestation (G7,5) and consequently in the density and organization of the mammary epithelium during lactation (L2: day 2 of lactation, 10: day 10 of lactation; +pups: in the presence of pups, −pups: in the absence of pups). Bar = 0.5 mm. Enlarged views of each developmental stage show the differences in mammary epithelial tissue organization between WT and *Sprn*^*0/0*^ mice. Bar = 200 nm.

We next investigated the potential incidence of *Sprn*^*0/0*^ invalidation on mammary gland development by whole mount analyses. The selected pictures showed in Fig. 6 are representative of the mammary morphology of at least 3 animals of both genotypes. In virgin, G7.5 and lactating MGs, some mammary primary canals were enlarged in *Sprn*^*0/0*^ tissues. In gestating and lactating tissues, *Sprn*^*0/0*^ MGs had less and only primary side branching compared to their wild-type counterparts and were overall less dense. This suggests that *Sprn*-knockout is associated with developmental defects.

To consolidate these observations, we explored whether Shadoo invalidation impacts proliferation and/or apoptosis of mammary epithelial cells. MG sections of each genotype were labeled for Ki67, active caspase3 at mid-gestation and full lactation (Fig. S4). At mid-gestation both proliferating and apoptotic cells were detected in developing alveoli from FVB/NJ MG. Double labeling with TUNEL indicated that aCasp3-positive apoptotic cells differed from Ki67-positive proliferating cells. In *Sprn*^*0/0*^ MGs, there were fewer proliferating Ki67-positive cells while apoptotic cells were found in similar or higher proportions than in WT MG. While apoptotic cells mostly localized near the forming lumen of alveoli in FVB/NJ, they appeared more dispersed in *Sprn*^*0/0*^ MG. During lactation, few proliferating and apoptotic cells were observed at the periphery of the alveoli in the mammary tissue of both FVB/NJ and *Sprn*^*0/0*^ mice. These results suggest that the absence of Shadoo generates an imbalance in favor of apoptosis in the growing mammary epithelial tissue, associated with a defect/delay in lumen formation.

### Sprn-invalidation affects mouse mammary transcriptomic

Comparative transcriptomic analysis was investigated on G7.5 WT and *Sprn*^*0/0*^ MGs. Fifty-three DEG were identified with a p-value < 0.05 and fc < −2 or >2, 21 downregulated and 32 upregulated (Table S5), including 8 downregulated small nuclear RNAs. Five differentially expressed genes were common between the placentas and the MGs (*SLC27A2, Snord13, Snord45b, Rnu3a, Snora73a*) and 9 between the embryos and the MGs (*AF357399, Hist2H2BF, MRP536, Ms4a4b, Snora23, Snora74a, Snora81, Rnu3a and Snora73a*), all of them behaving similarly between the different tissues, suggestive of shared but not yet identified pathways.

*Sprn*-knockout induces in the MG an immune response with the upregulation of 14 immunoglobulin genes, of CD209b and the downregulation of 2 MHC genes and 1 interferon inducible gene. IPA identified 3 networks (score > 17); Connective Tissue Development and Function, Humoral Immune Response, Infectious Disease (Table 5). It suggests that in *Sprn*^*0/0*^, gestating mammary epithelial cells undergo, associated with an immune reaction, a growth inhibition correlating with the whole mount observations. Search for deregulation of known regulators of mammary growth and branching morphogenesis revealed miR-145 overexpression in *Sprn*^*0/0*^ MGs. MiR-145 acts as an antitumor miRNA that targets Wnt5B whose overexpression is associated with mammary tumors^28^. Wnt5B was indeed downregulated in *Sprn*^*0/0*^ MGs but at a reduced fold-change (fc −1.4, p < 0.01). CIDEA, another potential regulator contributing to the control of mammary epithelial growth, is also upregulated (Table 6, Table S5). Transcriptional alteration of these genes is likely to contribute to epithelial growth inhibition. Furthermore, upregulation of both CIDEA and PPARGC1A (Table S5) may contribute to the observed difference in milk fat globules’ size distribution. GSEA analysis confirmed an abnormal proliferation of *Sprn*^*0/0*^ mammary tissue, highlighting a downregulation of genes related to protein localization to kinetochore (ESc4.593, FDR 2.75 10^−6^) and to DNA replication (ESc3.4, FDR 4.01 10^−8^).

**Table 5:** Identified networks with differentially expressed genes in G7.5 mammary glands.

Network	Differentially expressed genes in G7.5 mammary glands
Connective Tissue Development and Function	**CIDEA**, **COX7A1**, *H2AFZ*, *HLA-E*, **IgG**, **IGJ**, **IGKC**, **Igkv1–117**, **PHOSPHO1**, **PPARGC1A**, **SLC2A5**, *TPH1*,**UCP1**
Humoral Immune Response	*Apol9a/Apol9b*, *H2-T22*, *HLA-DRA*, *IFI16*, **IGHG1**, **Ighg2b**, **Igkv1–110**, **mir-145**, **Ms4a4b (includes others)**, **SLC27A2**
Infectious Disease	*BTG3*, **Cd209b**, **ELOVL3**, **F8**, **Igkv6–14**, **Iglv1**, **Retnlg**, **SLC4A4**

Bold-faced genes: positively differentially expressed genes in *Sprn0/0* mammary glands. Italized genes: negatively differentially expressed genes in *Sprn0/0* mammary glands.

**Table 6:** Summary of observed phenotypes and trancriptomic alterations in *Sprn*^*0/0*^ mice.

Reduced litter sizes			
Offspring		Phenotype	Transcriptomic
	Foetus (E7.5)	Early post-implantation increased lethality rate, growth retardation	Developmental disorder, cell-to-cell signaling and interaction, cell growth and proliferation, immunological and/or hematological disease, organismal survival
	Placenta (G14.5)	None detected	Lipid metabolism, molecular transport, small molecule biochemistry, tissue development and cellular movement
**Transient lactation deficit**			
**Mother**		**Phenotype**	**Transcriptomic**
	Mammary Gland	Reduced side branching, enlarged primary canals, altered mouse lactation, altered tissue development /morphology	Connective tissue development and function, humoral immune response, infectious disease
	Milk (L7.5)	Smaller milk fat globules	

Overall, transcriptomic analysis of *Sprn*^*0/0*^ gestating MG revealed the potential deregulations of pathways corroborating phenotypic and histologic analyses of this tissue. These results highlight a yet undescribed role of Shadoo in lactation.

## Discussion

The biological role of the members of the prion family remains mostly unknown, despite intensive researches focusing on PrP due to its major implication in TSE and potential involvement in other more prevalent neurodegenerative diseases such as Alzheimer’s disease. Less attention focused on the related Shadoo protein, probably resulting from its recent discovery and potentially limited implication in TSE^29,30^. The previously reported knockout of *Sprn* did not induce overt phenotypes beside a moderate, transient and sex-related, growth deficiency that could originate from a role of Shadoo in the control of feeding behavior, in association with its expression in hypothalamic neurons^12^.

A role of PrP in mouse embryonic development, around the implantation stage and the transition from anaerobic to aerobic metabolism, was suggested as well as in the development of the nervous system and of extra-embryonic tissues. Shadoo shares with PrP some biological properties^9^, although other biological roles are specific to this protein and/or even antagonist to PrP^20,21^. A biological redundancy between PrP and Shadoo was evocated following the observation that the knockdown of *Sprn* induces early embryonic lethality in *Prnp*^*0/0*^ mouse transgenic lines^10,11^. This hypothesis was weakened by the absence of phenotype of double *Prnp*/*Sprn* knockout mice^12^. The present report reinvestigates the role of Shadoo in FVB/NJ embryonic development through the generation of knockout mice using a ZFN approach, allowing its assessment in a non-mixed genetic background. This study highlights that *Sprn*^*0/0*^ mice suffer from subtle, sometimes transient, but significant alterations of developmental and differentiation processes that affect both embryonic and adult tissues (Table 6).

*Sprn*^*0/0*^ mice were viable and fertile, but analysis of the transmission rate in heterozygous intercrosses and of the average litter size in knockout homozygous crossings revealed a non-Mendelian distribution of the genotypes and a reduced number of pups, respectively. Further investigations highlighted an abnormal lethality rate of *Sprn*^*0/0*^ embryos at an early post implantation developmental stage, between E4.5 and E6.5. This suggested that the absence of Shadoo is not detrimental for embryonic implantation but weakens the survival potential of the embryos early post-implantation.

Absence of Shadoo transiently also affects the growth rate of the pups in this genetic background but independently from their sexes. This slightly differs from previous observations^12^. The difference could originate from the genetic backgrounds used in relation to the invalidation strategies. Although not formally exclusive with an abnormal feeding behavior, this transient phenotype could correlate with an abnormal biology of the placentas, as judged by our transcriptomic studies (Table 6). Invalidation of *Sprn* led to placental defects, recalling observations made following *Prnp*-knockout^14^. However, in *Sprn*^*0/0*^ mice, no histological placental defect were evidenced and the few genes described as DEG in *Prnp*^*0/0*^ placentas were not transcriptionally affected in *Sprn*^*0/0*^. Our transcriptomic analysis rather suggests that *Sprn*^*0/0*^ extra-embryonic tissues might undergo early developmental defects, highlighted by transcriptomic alteration in E7.5 embryos of genes affecting the formation of mouse extra-embryonic tissues such as Smad1. These early potential defects seem at least partially corrected during development, as exemplified by the activation of pathways at G14.5 supporting placentation and embryonic development. Studies of earlier developmental stages to assess placenta development should help validating this hypothesis.

We also evidenced an impact of the absence of Shadoo on MG physiology, objectivized by (i) growth curve analyses of FVB/NJ pups fed by *Sprn*^*0/0*^ females, (ii) transcriptomic alterations of G7.5 mammary tissue, (iii) morphological development defects and alteration of milk fat globules synthesis that correlate with transcriptomic alterations, (iv) mammary cell/membrane alterations. Implication of the Prion protein family in the physiology of the MG per se has received little attention. Implication of PrP in mammosphere formation and repopulating activity was documented^25^, but its relationship with lactation performances remains controversial. Of note is the absence of any mammary phenotype described so far for *Prnp*^*0/0*^ mammals. In the present study, invalidation of Shadoo appears to induce various gestational/lactation mammary tissue and cellular defects that results in transient lactation deficit.

This phenotype exemplified Shadoo implication in the control of cellular proliferation and differentiation, also revealed at early post-implantation embryonic stages. Such an involvement in cell proliferation and differentiation was highlighted for PrP^31^. Implication of these two related proteins in similar biological functions alongside their expression in developing mammalian fetuses could substantiate the hypothesis of their biological redundancy. Surprisingly, our data rather suggest that *Sprn* and *Prnp* exert opposite effects on expression of master regulators of pluripotency and/or embryonic development, at least at E7.5. Thus, one could expect their co-invalidation to be neutral, as documented^12^. However, comparison of the data described here and for *Sprn*-knockdown/*Prnp*^*0/0*^ embryos^10,11^ highlights a developmental delay in the embryonic lethality between *Sprn*^*0/0*^ and *Sprn*-knockdown/*Prnp*^*0/0*^ embryos. Lethality of *Sprn*^*0/0*^ embryos occurs early after implantation while that of *Sprn*-knockdown/*Prnp*^*0/0*^ embryos was evidenced only at E10.5^10^, with E7.5 decidua carrying abnormal embryos^11^. It could suggest that *Sprn*-knockdown induces a fragility of the embryos at *implantation that becomes detrimental to the further development of the Prnp*^*0/0*^ *ones*, between E8.5 and E9, *when* upturn of PrP RNA expression should occur^32^. Absence of such a scenario in *Prnp*^*0/0*^/*Sprn*^*0/0*^ mice might result from genetic adaptation^13^.

In conclusion, this study provides evidence for a yet controversial role of the Prion-related protein Shadoo during mouse embryogenesis and more generally in the control of cellular proliferation and differentiation of various tissues, including adult ones such as the mammary epithelium. It also highlights a potential embryonic antagonist action of PrP and Shadoo, revisiting alongside recent papers the current view of a potential biological redundancy between these two proteins.

## Methods

### Ethics statement

All animal experiments were carried out in strict accordance with the recommendations in the guidelines of the Code for Methods and Welfare Considerations in Behavioral Research with Animals (Directive 2016/63/UE). All efforts were made to minimize suffering. Experiments were approved by the INRAE local animal experiment ethics committee of Jouy-en-Josas (Comethea, Permit Number 02532.01). All animal manipulations were done according to the recommendations of the French Haut Conseil aux Biotechnologies, HCB (Permit Number N°6461 and DUO N°5468).

### Generation and establishment of Sprn-knockout mice

A preparation of mRNA encoding a ZFN targeting the start of the *Sprn* ORF was purchased from Sigma-Aldrich Corp (CompoZrTM ZFN design). The ZFN target site was 5′-ctgtgacagctgttccgccangggcggccgcggaggcg-3′. ZFN mRNA was injected at 2 ng/μl into 1 cell-fertilized FVB/NJ mouse eggs. Surviving injected eggs were transferred into pseudo-pregnant recipient mice. Tail-DNA analysis of the 29 resulting live pups was performed by PCR using oligonucleotides 5′-gccggccttacgcgtactcttga-3′ and 5′-gcctattcccaagccctgatac-3′, using the Promega GoTaq G2 Flexi DNA polymerase kit. PCR conditions were made of 40 amplifications cycles; 94 °C-30 s, 60 °C-30 s and 72 °C-30 s. The amplified 400 bp long DNA genomic region surrounding the ZFN target site was sequenced to search for putative mutations.

Transgenic founder mice were crossed with FVB/NJ mice to establish transgenic lines. Intercrosses between heterozygous mice were used to derive transgenic knockout lines.

### Reproductive performance analyses

All examined females were on their first pregnancy or lactation. Intercrosses between heterozygous mice and between homozygous mice were performed and the number and genotype of pups recorded. To assess embryonic lethality, pregnant FVB/NJ or *Sprn*^*0/0*^ females, following mattings with males of the same genotype, were euthanized at different gestation stages. Implantation points were recorded. Development and vitality of the embryos were observed under binocular microscope.

Comparative analysis of growth curves of FVB/NJ and *Sprn*^*0/0*^ pups was performed, using FVB/NJ lactating females as foster mothers. Lactation performance was indirectly assessed by analysis of growth curves of FVB/NJ pups fed either by FVB/NJ or *Sprn*^*0/0*^ females. Six pups were placed under each lactating females and 3 females in their first lactation of each genotype analyzed. Milk samples were collected at L7.5 on other lactating females following pups removal for 3 hours and 300 µl −10 UI intra-peritoneal injection of oxytocin.

### RNA purifications and RT-(q)PCR

Total RNA was isolated from (i) E7.5 mouse embryos, (ii) E14.5 placentas and (iii) G7.5 MGs (first pregnancy). Six gestating females of each genotype were used at each analyzed stages. Individual RNA extractions were performed using the RNeasy Lipid Tissue Mini kit (Qiagen cat # 75842). RNA concentration was calculated by electro-spectrophotometry and the RNA integrity checked with the Agilent Bioanalyser (Waldbroom, Germany). All extracted RNA samples had a RIN value > 8.5.

Reverse transcription was performed on 5μg of total RNA, using Invitrogen SuperScript^TMIII^ reverse transcriptase kit (18080051) or RTVilo (11754050) and random primers, according to the manufacturer’s instructions. Amplification of the mouse *Sprn* cDNA ORF was performed using oligonucleotides 5′-taggcttgtgaccaattcttgcc-3′ and 5′-ggagtttagcctggtctaaggc-3′, that are located on the two separate *Sprn* exons to avoid amplification of potential contaminating genomic DNA. PCR conditions were made of 40 amplifications cycles; 94 °C-30 s, 60 °C-60 s and 72 °C-30 s, using GC-rich adapted PCR amplification kit (KAPA2G Robust PCR kit, SigmaAldrich). Two WT and two *Sprn*^*0/0*^ mouse brains were analyzed.

For RT-qPCR, quantification was performed on triplicates, using 3 different RNA samples for each genotype, using the ABsolute Blue QPCR SYBR Green ROX Mix (Thermo Scientific) and standard PCR conditions. Primers were designed on separate exons, using Primer 3 software (http://primer3.ut.er) to produce 100-bp amplicons, with a Tm of 60 °C. The TBP and SDHA genes were used for normalization, using primers 5′-ttcgtgcaagaaatgctgaa-3′ and 5′-tcctgtgccacaccatttttc-3′ for TBP and 5′-aaggacctggcatcaagaga-3′ and 5′-tgatctttctcagggccaca-3′ for SDHA. Tested genes were α1-antitrypsin, using primers 5′-ctccggaatcacagaggaaa-3′ and 5′-gcttctgttcctgtctcatcg-3′, GC using primers 5′-gcaaagacctctgtggtcag-3′ and 5′-agggttggctccagaacttt-3′, Gli3 using primers 5′-tcccacgagaacagatgtca-3′ and 5′-tgaggctgcatatgtattgc-3′, TBPA using primers 5′-tgaagagctgaaccactgga-3′ and 5′-ccaggcataggatgactagga-3′ and PSG23 using primers 5′tcacctggaaagacactgga-3′ and 5′-ggcagagtcaagagggtcac-3′.

### Western blot analysis

Following mouse cervical dislocation, FVB/NJ and *Sprn*^*0/0*^ mouse brains were crushed in 0.32 M Sucrose solution containing cocktail of protease inhibitors (Roche) and enriched in subcellular membranes by ultra-centrifugation (150 000 × g) as developed in Westaway *et al*. (2011). Proteins were extracted from membrane pellets by N-PER Neuronal Protein reagent (Thermo). Amounts of 50 μg sample proteins were separated on a home-cast 14% polyacrylamide gel SDS-PAGE and were transferred on PVDF membrane by the Trans-blot Turbo (Bio-Rad). Non-specific reactivity was blocked by incubation for 1 h with a 5% skimmed milk in 0.1% TBS-Tween 20 [TBS-T]. Blot was incubated with a serum from a rabbit immunized against mouse Shadoo, obtained from Covalab (https://www.covalab.com/), at 1/750e dilution in 0.1% TBS-T for two hours at room temperature or with other antibodies directed against Shadoo that are listed in the text at recommended dilutions. Horseradish peroxidase-conjugated anti-IgG from various origins according to the primary antibody were used as secondary antibody with 1 h incubation. Excess first and second antibodies were removed by washing 3 × 10 min in TBS-T buffer. Detection was accomplished with a chemiluminescence system (ECL Prime, GE Healthcare) and exposure in ChemiDoc Touch device (Bio-Rad).

Fibrinogen and α1-antitrypsin protein ratio between wild type and *Sprn*^*0/0*^ mouse were estimated in placenta samples with Image Lab software 5.2 (BioRad). They were crushed with ultra-turrax in N-PER Neuronal Protein reagent containing cocktail protease inhibitors (Roche). The experimental protocol was followed as described above according recommended factor dilutions, using Abcam ab189490 and Abcam ab231093 antibodies, respectively.

### Milk collect and analysis

Milk was collected from L7.5 *Sprn*^0/0^ and FVB/NJ females on their first lactation. Milk protein concentration was estimated using Bradford’s procedure and fractionation of its proteins was performed on home-cast SDS-PAGE 12.5% polyacrylamide followed by coomassie blue staining. A western blot was performed as described above with a special antibody designed against all mouse milk proteins (009 RAM/MSP Nordic Immunology).

### Milk fat globules (MFGs) labeling, image acquisition and analysis

Experiments involved analyses of at least 3 mice per genotype and condition. Whole mouse milk (L10) was diluted 1:4 in sterile water and fixed with PBS containing 4% paraformaldehyde (PFA) for 10 minutes at 4 °C, as described^26^. Milk fat globules (MFGs) were then labeled with acridine orange (AO, 0.1%), Alexa594-conjugated wheat germ agglutinin (WGA, 1 mg/ml), or filipin (1 mg/ml) diluted in PBS for 30 min at 4 °C. Neutral lipids were concomitantly counterstained with BODIPY 493/503 (3 µg/ml) or Nile Red (1 µg/ml). 50 µl of each stained sample was mixed with 50 µl of 1% low melt agarose solution, placed on a glass slide and topped with a coverslip. Fluorescence microscopy was performed with an Axio observer Z1 microscope (Zeiss) equipped with standard filters for FITC, Rhodamine and DAPI emissions, using a 40x objective and a Greyscale CCD camera CoolSnap HQ2 camera (Photometrics) coupled to the Axiovision imaging system software. About thirty pictures of each condition were randomly acquired for each condition and both the number and the perimeter (µm) of the MFGs were estimated using ImageJ (1.47q software 2012, http://rsb.info.nih.gov/ij/). The volume of the MFGs (µm^3^) were calculated and a Kruskall-Wallis rank test was performed using the version 3.5.2 of the R Software (R core Team 2018).

### Placenta histological analysis

Immediately after dams’ sacrifice by cervical dislocation at G14.5 or G17.5, the uterus were removed and the placentas collected. Intact placentas (6 per genotype and stage from 3 different dams) were fixed in formalin (48 hours). After fixation, each placenta was bisected adjacent to the umbilical vessels. Both halves were embedded in paraffin. Section (3–5 µM) from each placenta were processed for Hematoxylin-Eosin coloration and examined by a qualified veterinary pathologist.

### MG whole mount and immunohistological analyses

Experiments involved analyses of at least 3 mice per genotype and condition. Inguinal MGs (4^th^, left) were excised, spread on glass slides and fixed in Carnoy’s fixative (100% EtOH, chloroform, glacial acetic acid; 6∶3∶1) for 4 h at room temperature, washed in 70% EtOH for 15 min, rehydrated in water, and stained in carmine alum (2% carmine and 5% aluminum potassium sulfate in water) overnight. Tissues were then gradually dehydrated through serial ethanol baths and cleared in xylene overnight. MGs were kept in Permount Mounting Media (Fisher Scientific) until images were captured.

For immunohistological analyses, mouse MGs were collected at different physiological stages and processed as described^26^. Tissue sections were labeled using Alexa488-conjugated WGA, rhodamine-conjugated phalloidin (Thermo Fisher), or filipin (Sigma-Aldrich) diluted in PBS for 30 min at 4 °C. Neutral lipids and nuclei were counterstained with BODIPY 493/503 or Nile Red and DAPI (4′-6-diamidino-2-phenylindole), respectively. Slides were mounted with Vectashield (Vector Laboratories LTD, United Kingdom) and stored at 4 °C until observation. For some experiments, Tissue sections or MFGs were also labeled for actin with rhodamine-conjugated phalloidin (Molecular Probes), GM1 ganglioside with Alexa 594–conjugated CTxB (Invitrogen), or free CH using filipin (Sigma-Aldrich). Each experiment was performed at least twice and included negative control without primary antibody. At least 20 images were randomly acquired per condition tested and further analyzed. All images were analyzed using ImageJ 1.47q software (http://rsb.info.nih.gov/ij/).

### Transcriptomic analyses

Transcriptomic analyses were performed using Affymetrix MouseGene 2,OSt microarray on the Institute Cochin Genom’ic platform. Each analyzed embryonic RNA sample derived from the equal mixing of 6 individual total RNA preparations from E7.5 embryos. Different embryonic samples were used for each mixture. Similarly, each analyzed placenta RNA sample derived from the equal mixing of 6 individual total RNA preparations from E14.5 placentas. Different individual samples were used for each mixture. Each MG RNA sample derived from the equal mixing of 3 individual total RNA preparations from G7.5 MGs. Different individual samples were used for each mixture. Three biological replicates per tissue and genotype were performed. Differentially expressed genes between FVB/NJ and FVB/NJ *Sprn-*knockout tissues were identified following normalization of the raw data, with fold chance ratios and p values determined as described in the results section. Data were analyzed by Gene set enrichment analysis (GSEA, http://software.broadinstitute.org/gsea/index.jsp). Differentially expressed genes were clustered and classified in pathways and networks by using Ingenuity (http://www.ingenuity.com/).

## Supplementary information

Supplementary information.
