# Supplementary material for: The Prion-like protein Shadoo is involved in mouse embryonic and mammary development and differentiation
Source: Sci Rep. 2020 Apr 21;10:6765. doi: 10.1038/s41598-020-63805-y (PMC7174383; doi:10.1038/s41598-020-63805-y)
Supplement: Supplementary file 1 — Supplementary information. [file 41598_2020_63805_MOESM1_ESM.pdf]

Supplementary information

The Prion-like protein Shadoo is involved in mouse embryonic and mammary development and differentiation.

Bruno Passet, Johan Castille, Samira Makhzami, Sandrine Truchet, Anne Vaiman, Sandrine Floriot, Katayoun Moazami-Goudarzi, Marthe Vilotte, Anne-Laure Gaillard, Louise Helary, Maud Bertaud, Olivier Andréoletti, Daniel Vaiman, Pierre Calvel, Nathalie Daniel-Carlier, Mohammed Moudjou, Christian Beauvallet, Mohamed Benharouga, Denis Laloé, Sophie Mouillet-Richard, Amandine Duchesne, Vincent Béringue, Jean-Luc Vilotte

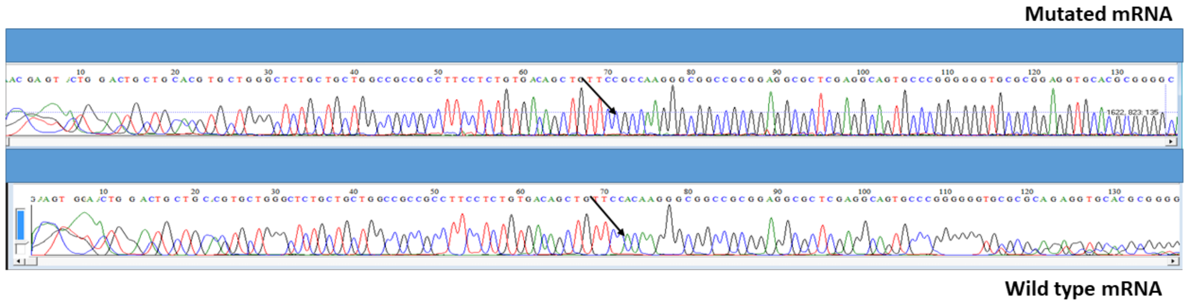

Supplementary Figure 1: Partial sequence analysis of *Sprn* transcripts.

Comparative sequence analysis of WT and mutated (line 17) *Sprn* transcripts following RT-PCR. The arrow indicates the location of the mutation (deletion of 1 C nt and A to G mutation of the adjacent one).

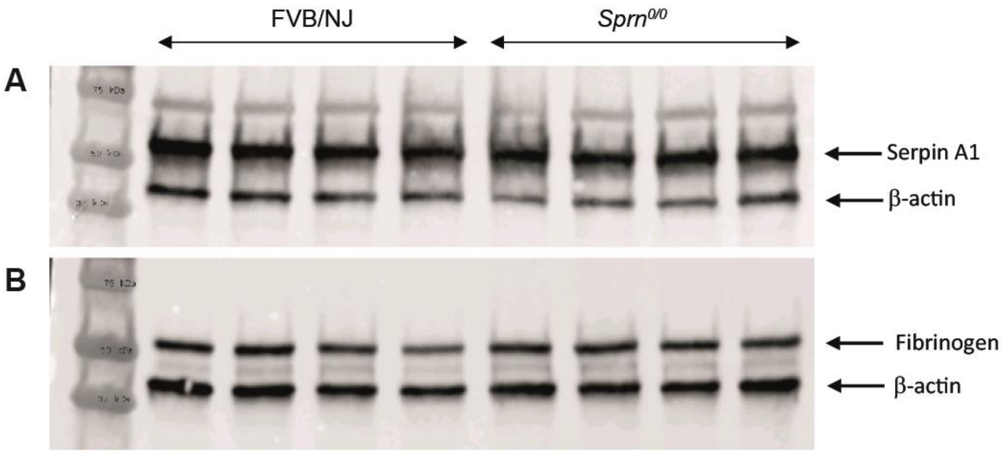

Relative value	FVB/NJ	*Sprn*$^{0/0}$
SerpinA1	2.97 ± 0.38	4.12 ± 0.91
Fibrinogen	0.59 ± 0.06	0.72 ± 0.04

Supplementary Figure 2: Placental SepinA1 and Fibrinogen comparative analysis.

A. Comparative western analysis of placental SerpinA1 protein levels between FVB/NJ and *Sprn*$^{0/0}$ mice following SDS-PAGE. Origin of the samples is indicated in the top of the gel. Left lane: molecular weight marker. Right margin: identified proteins.

B. Comparative Western analysis of placental Fibrinogen protein levels between FVB/NJ and *Sprn*$^{0/0}$ mice following SDS-PAGE. Origin of the samples is indicated in the top of the gel. Left lane: molecular weight marker. Right margin: identified proteins.

Table: relative protein levels between FVB/NJ and *Sprn*$^{0/0}$ placentas. These data are derived from the above westerns using the Image Lab software (BioRad) and β-actin as an internal loading control (that was not detected as differentially expressed in the micro-array analysis).

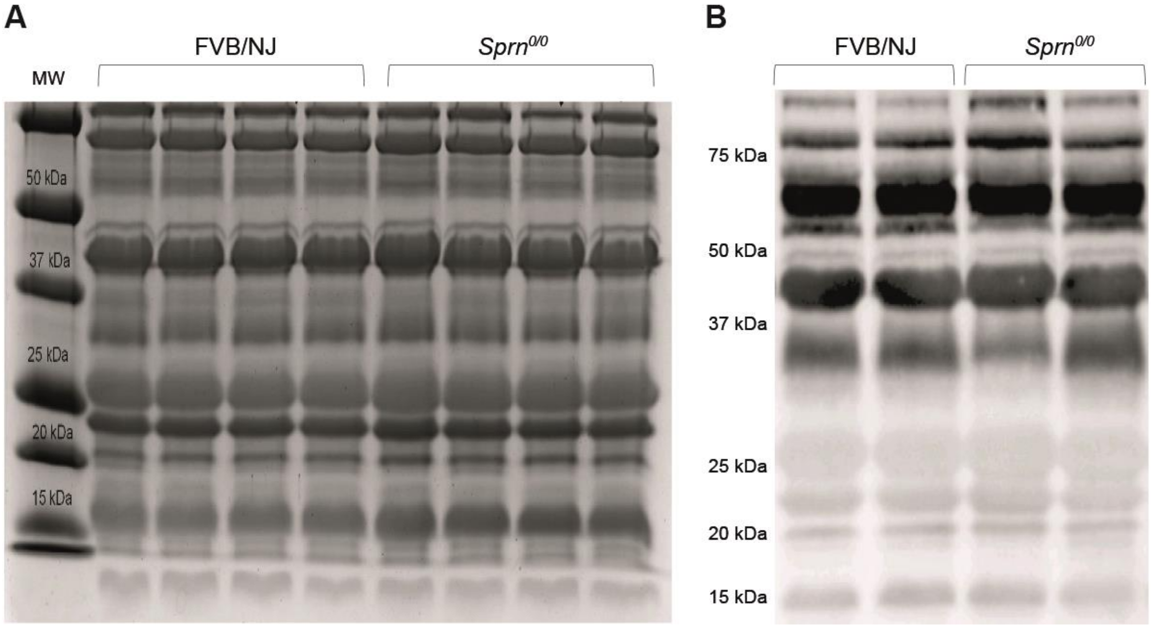

Supplementary Figure 3: Protein milk comparative analysis.

A. Comparative analysis of milk protein composition between WT (FVB/NJ) and *Sprn*$^{0/0}$ mice following SDS-PAGE and coomassie blue staining. Origin of the samples is indicated in the top of the gel. Left lane (MW) and margin: molecular weight marker.

B. Western analysis of major milk proteins, using the 009 RAM/MSP (Nordic Immunology) antibody. Left margin: molecular weight marker.

No significant difference could be detected between WT and *Sprn*$^{0/0}$ mouse milk samples.

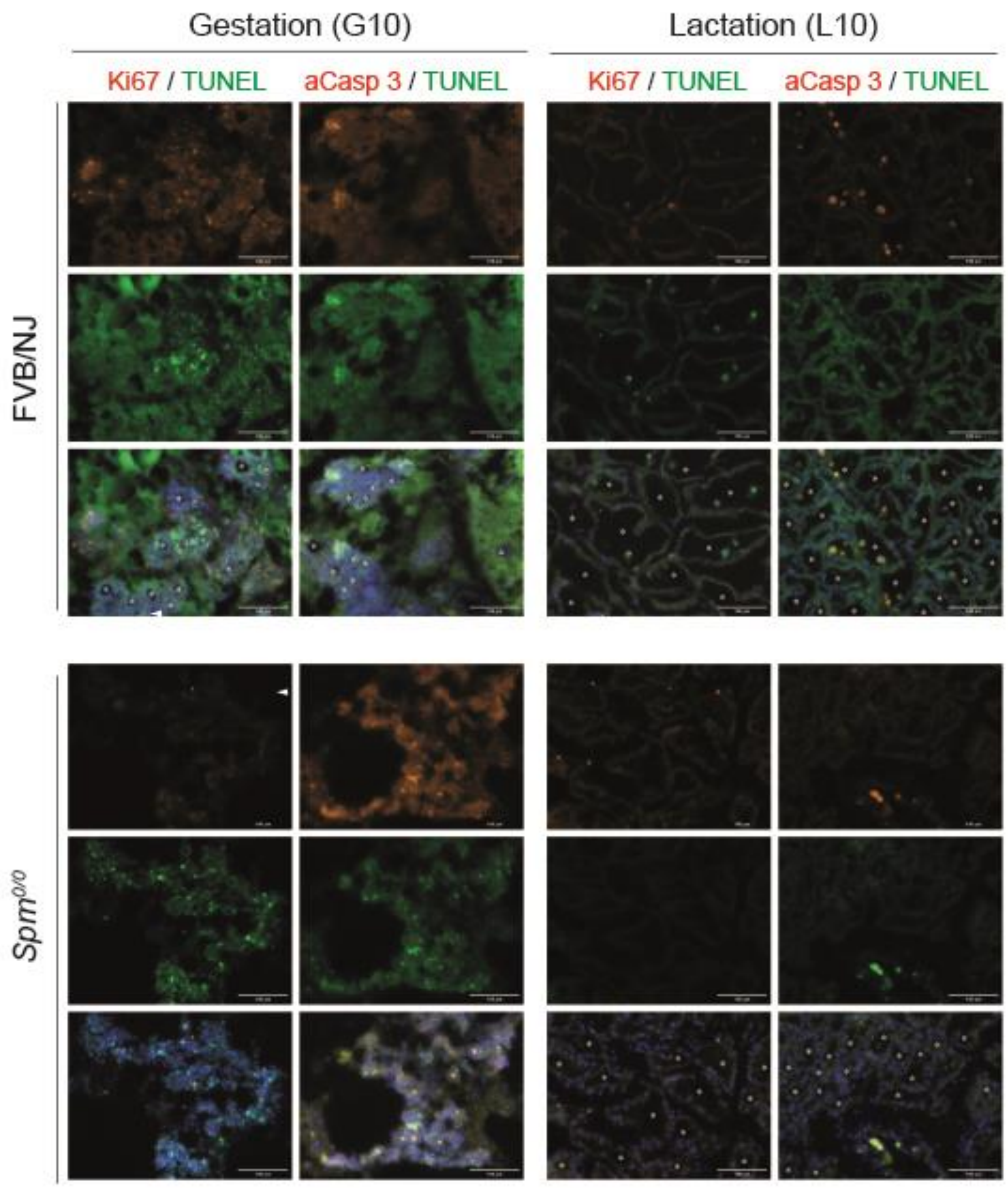

Supplementary Figure 4: Balance analysis between proliferation and apoptosis in the mammary gland

Confocal microscopy imaging of mammary gland sections from FVB/NJ and *Sprn*$^{0/0}$ mice at mid-gestation (G10) or mid-lactation (L10) co-strained for Ki67 (red), active caspase-3 (aCasp3, red), and/or by TUNEL (green), to detect proliferating and apoptotic cells, respectively. Nuclei were counterstained with nuclear marker 4',6-diamidino-2-phenylindole (DAPI, blue). The Asterisks indicate the lumen Bar, 100µm.

Supplementary Note 1: Further transcriptomic analysis of E7.5 $Sprn^{0/0}$ embryos.

We investigated if known regulators of EMT and/or stem cell fate maintenance were differentially expressed in $Sprn^{0/0}$ E7.5 but at a reduced fold change (less than 2 fold). Several master regulators of pluripotency were indeed affected with the down-regulations of miR-27B (fc -1.98, p<0.02)[1], Oct4 (fc -1.5, p<0.01)[2], Smad1 (fc -1.2, p<0.005)[3], Wnt1 (fc -1.33, p<0.01)[4] and the upregulation of TGFβ receptors (TGFβr2, fc + 1.6, p<0.003 and TGFβr3, fc +1.4, p<0.007)[5], Snai2 and BMP4 (fc +1.6, p<0.015 and +1.6, p<0.0015 respectively)[6].

Another deregulated pathway involved the downregulation of miR-15A and miR-16.2 (fc -1.42, p<0.02) and the associated upregulation of SLC6A4 that could affect embryo development and viability through serotonin control of cell proliferation, migration and differentiation[7]. In line with these pathways is the observed upregulation of 6 members of the SLC39a ZINC transporters protein family (SLC39a10, SLC39a1, SLC39a6, SLC39a14, SLC39a9 and SLC39a8, p<0.05, fc between 1 and 2), of 3 cadherin-encoding genes (Cdh3, Cdh4 and Cdh5) and of 6 integrin-encoding genes (Itga1, Itga9, Itga2, Itgav, Itga6 and Itgb5, supplementary Table1).

1. Crist CG. et al. Muscle stem cell behavior is modified by microRNA-27 regulation of Pax3 expression. Proc Natl Acad Sci U S A. **106**(32), 13383-13387. (2009).

2. Sajini AA, Greder LV, Dutton JR, Slack JM. Loss of Oct4 expression during the development of murine embryoid bodies. Dev Biol. **371**(2), 170-179. (2012).

3. Suzuki A. et al. Nanog binds to Smad1 and blocks bone morphogenetic protein-induced differentiation of embryonic stem cells. Proc Natl Acad Sci U S A. **103**(27), 10294-10299. (2006).

4. Aubert J, Dunstan H, Chambers I, Smith A. Functional gene screening in embryonic stem cells implicates Wnt antagonism in neural differentiation. Nat Biotechnol. **20**(12), 1240-1245. (2002).

5. Li R. et al. A mesenchymal-to-epithelial transition initiates and is required for the nuclear reprogramming of mouse fibroblasts. Cell Stem Cell. **7**(1), 51-63. (2010).

6. Richter A. et al. BMP4 promotes EMT and mesodermal commitment in human embryonic stem cells via SLUG and MSX2. Stem Cells. **32**(3), 636-48. (2014).

7. Moya PR, Wendland JR, Salemme J, Fried RL, Murphy DL. miR-15a and miR-16 regulate serotonin transporter expression in human placental and rat brain raphe cells. Int J Neuropsychopharmacol. **16**(3), 621-629. (2013).

Supplementary Note 2: Further transcriptomic analysis of E7.5 *Sprn*$^{0/0}$ placentas.

We investigated whether upstream regulators supporting observed transcriptomic alterations and/or placentation and embryonic development are present in differentially expressed genes with reduced fold change. It is indeed consistent with the observed deregulation of upstream regulators such as the upregulation of both *Hnf-1*β (fc +1.6, $p<0.008$) and *Hnf-4*α (fc +1.6, $p< 0.005$) and the downregulation of *Pkd1* (fc -1.19, $p<0.07$)[1,2]. Of note, is the observed upregulation of TPH1 (fc +1.6, $p<0.01$) involved in serotonin metabolism, highlighting again a potential link between Shadoo and serotonin. Expression of genes involved in serotonin metabolism were described in mouse placenta[3] in relation with the control exerted by placental serotonin on forebrain development[4].

1. Allen E. et al. Loss of polycystin-1 or polycystin-2 results in dysregulated apolipoprotein expression in murine tissues via alterations in nuclear hormone receptors. Hum Mol Genet. **15**(1):11-21. (2006).

2. Rebholz SL, Burke KT, Yang Q, Tso P, Woollett LA. Dietary fat impacts fetal growth and metabolism: uptake of chylomicron remnant core lipids by the placenta. Am J Physiol Endocrinol Metab. **301**(2), E416-425. (2011).

3. Wu HH, Choi S, Levitt P. Differential patterning of genes involved in serotonin metabolism and transport in extra-embryonic tissues of the mouse. Placenta. **42**, 74-83. (2016).

4. Bonnin A, Goeden N, Chen K, Wilson ML, King J, Shih JC, Blakely RD, Deneris ES, Levitt P. A transient placental source of serotonin for the fetal forebrain. Nature. **472**(7343), 347-350. (2011).

Supplementary Table 1: Differentially expressed genes in E7.5 *Sprn*$^{0/0}$ embryos

Following normalization of the raw data, differentially expressed genes with a p-value <0.05 and a fold change (fc) inferior to -2 or superior to 2 were selected and are listed.

Fold Change	Symbol	Entrez Gene Name	Location	Type(s)	p-value
2.168	1600015I10Rik	RIKEN cDNA 1600015I10 gene	Other	other	1.16E-03
-3.731	AF357399	snoRNA AF357399	Other	other	5.94E-03
2.075	ALG12	ALG12. alpha-1.6-mannosyltransferase	Cytoplasm	enzyme	5.05E-04
2.207	AMPD3	adenosine monophosphate deaminase 3	Cytoplasm	enzyme	4.92E-03
2.175	ANGPT2	angiopoietin 2	Extracellular Space	growth factor	2.40E-03
2.350	ANXA11	annexin A11	Nucleus	other	1.10E-04
3.263	AOC1	amine oxidase. copper containing 1	Extracellular Space	enzyme	7.47E-04
-2.149	ATP6V1G1	ATPase. H+ transporting. lysosomal 13kDa. V1 subunit G1	Cytoplasm	transporter	2.18E-03
3.509	BIN2	bridging integrator 2	Plasma Membrane	other	1.18E-02
2.678	C16orf58	chromosome 16 open reading frame 58	Other	other	1.35E-04
2.334	C1S	complement component 1. s subcomponent	Extracellular Space	peptidase	3.21E-03
2.825	C3	complement component 3	Extracellular Space	peptidase	1.49E-02
2.655	C5AR2	complement component 5a receptor 2	Plasma Membrane	G-protein coupled receptor	1.02E-03
2.177	CASP8	caspase 8. apoptosis-related cysteine peptidase	Nucleus	peptidase	6.24E-04
2.351	CAT	catalase	Cytoplasm	enzyme	1.69E-06
2.148	CAV1	caveolin 1. caveolae protein. 22kDa	Plasma Membrane	transmembrane receptor	1.65E-05
2.147	CD68	CD68 molecule	Plasma Membrane	other	1.24E-03
-2.240	CHCHD1	coiled-coil-helix-coiled-coil-helix domain containing 1	Nucleus	other	5.06E-04
2.082	CLEC1A	C-type lectin domain family 1. member A	Plasma Membrane	transmembrane receptor	6.34E-06
2.283	COQ5	coenzyme Q5 homolog. methyltransferase (S. cerevisiae)	Cytoplasm	enzyme	1.40E-04
2.056	CRCT1	cysteine-rich C-terminal 1	Other	other	1.14E-02
2.064	Cyb5r3	cytochrome b5 reductase 3	Other	enzyme	5.40E-05
2.503	CYBB	cytochrome b-245. beta polypeptide	Cytoplasm	enzyme	7.94E-04

2.037	DAG1	dystroglycan 1 (dystrophin-associated glycoprotein 1)	Plasma Membrane	transmembrane receptor	1.39E-05
4.885	DCN	decorin	Extracellular Space	other	2.50E-07
2.045	DES	desmin	Cytoplasm	other	3.99E-02
2.677	DHRS9	dehydrogenase/reductase (SDR family) member 9	Cytoplasm	enzyme	6.83E-05
-2.056	DQ267100	snoRNA DQ267100	Other	other	4.94E-03
2.506	Ear2 (includes others)	eosinophil-associated. ribonuclease A family. member 2	Cytoplasm	enzyme	3.76E-02
2.009	EHD2	EH-domain containing 2	Nucleus	other	7.05E-04
2.967	EHD3	EH-domain containing 3	Cytoplasm	other	9.95E-03
2.511	Erv3	endogenous retroviral sequence 3	Other	other	8.93E-03
2.253	FABP4	fatty acid binding protein 4. adipocyte	Cytoplasm	transporter	2.40E-02
2.219	FADS3	fatty acid desaturase 3	Plasma Membrane	enzyme	2.76E-03
2.405	FERMT1	fermitin family member 1	Plasma Membrane	other	1.51E-06
2.075	FSTL3	follistatin-like 3 (secreted glycoprotein)	Extracellular Space	other	2.75E-03
2.409	GDPGP1	GDP-D-glucose phosphorylase 1	Cytoplasm	other	2.29E-03
2.782	GLP1R	glucagon-like peptide 1 receptor	Plasma Membrane	G-protein coupled receptor	8.70E-05
-2.183	Gm12238	predicted gene 12238	Other	other	1.17E-03
4.025	Gm6445	predicted gene 6445	Other	other	7.95E-06
2.411	GPX3	glutathione peroxidase 3 (plasma)	Extracellular Space	enzyme	1.92E-03
2.105	GREM2	gremlin 2. DAN family BMP antagonist	Extracellular Space	other	5.72E-05
2.233	Gstm3	glutathione S-transferase. mu 3	Cytoplasm	enzyme	1.93E-04
2.396	GZMH	granzyme H (cathepsin G-like 2. protein h-CCPX)	Cytoplasm	peptidase	1.98E-03
2.173	HEATR2	HEAT repeat containing 2	Other	other	3.11E-05
-2.038	HIST2H2BF	histone cluster 2. H2bf	Nucleus	other	1.02E-02
5.062	HSD3B1	hydroxy-delta-5-steroid dehydrogenase. 3 beta- and steroid delta-isomerase 1	Cytoplasm	enzyme	7.36E-06
-2.324	HSPA1A/HSPA1B	heat shock 70kDa protein 1A	Cytoplasm	enzyme	8.96E-03
-2.301	Hspa1b	heat shock protein 1B	Cytoplasm	other	1.78E-03

2.358	HSPB7	heat shock 27kDa protein family. member 7 (cardiovascular)	Cytoplasm	other	1.10E-03
2.119	IFI44	interferon-induced protein 44	Cytoplasm	other	3.96E-02
2.001	IGSF11	immunoglobulin superfamily. member 11	Plasma Membrane	other	2.05E-03
2.362	IGSF6	immunoglobulin superfamily. member 6	Plasma Membrane	transmembrane receptor	1.39E-03
2.013	IRAK3	interleukin-1 receptor-associated kinase 3	Cytoplasm	kinase	7.07E-03
2.068	ITGA2	integrin. alpha 2 (CD49B. alpha 2 subunit of VLA-2 receptor)	Plasma Membrane	transmembrane receptor	4.29E-04
2.145	LAPTM5	lysosomal protein transmembrane 5	Plasma Membrane	other	2.15E-02
2.146	LILRB4	leukocyte immunoglobulin-like receptor. subfamily B (with TM and ITIM domains). member 4	Plasma Membrane	other	9.62E-03
-2.078	LSM3	LSM3 homolog. U6 small nuclear RNA associated (S. cerevisiae)	Nucleus	other	5.78E-04
2.383	LY96	lymphocyte antigen 96	Plasma Membrane	transmembrane receptor	7.75E-03
-2.073	MALAT1	metastasis associated lung adenocarcinoma transcript 1 (non-protein coding)	Nucleus	other	8.99E-05
-2.238	Mettl7a2/Mettl7a3	methyltransferase like 7A3	Other	other	2.63E-02
2.152	MFSD2A	major facilitator superfamily domain containing 2A	Plasma Membrane	transporter	5.35E-04
-3.185	mir-15	microRNA 15a	Cytoplasm	microRNA	9.50E-06
-2.702	mir-17	microRNA 17	Cytoplasm	microRNA	2.76E-06
-3.093	mir-19	microRNA 19a	Cytoplasm	microRNA	1.82E-04
-2.121	mir-302	microRNA 302c	Cytoplasm	microRNA	3.78E-03
-2.188	mir-3070	microRNA 3070b	Cytoplasm	microRNA	2.81E-03
-2.857	mir-467	microRNA 466	Other	microRNA	3.73E-04
-2.186	mir-665	microRNA 665	Cytoplasm	microRNA	1.37E-04
-2.331	mir-677	microRNA 677	Cytoplasm	microRNA	2.48E-04
-2.124	mir-679	microRNA 679	Cytoplasm	microRNA	3.01E-03
-2.508	MIR17HG	miR-17-92 cluster host gene (non-protein coding)	Other	other	5.58E-04
-2.442	Mir3080	microRNA 3080	Cytoplasm	microRNA	4.29E-05
-2.003	Mir5112	microRNA 5112	Cytoplasm	microRNA	4.15E-02

2.962	Mocs1	molybdenum cofactor synthesis 1	Cytoplasm	enzyme	1.54E-05
2.493	MPEG1	macrophage expressed 1	Cytoplasm	other	8.19E-06
2.078	MRPS36	mitochondrial ribosomal protein S36	Cytoplasm	other	4.87E-02
2.109	Ms4a4b (includes others)	membrane-spanning 4-domains. subfamily A. member 4B	Plasma Membrane	other	4.25E-02
2.294	MUC13	mucin 13. cell surface associated	Extracellular Space	other	2.17E-04
-2.166	n-R5s136	nuclear encoded rRNA 5S 136	Other	other	1.96E-05
-2.179	Neat1	nuclear paraspeckle assembly transcript 1 (non-protein coding)	Nucleus	other	7.64E-04
2.024	NFYA	nuclear transcription factor Y. alpha	Nucleus	transcription regulator	9.41E-05
2.005	NSUN3	NOP2/Sun domain family. member 3	Other	other	3.43E-04
2.191	OSMR	oncostatin M receptor	Plasma Membrane	transmembrane receptor	3.57E-04
2.505	PAPSS2	3'-phosphoadenosine 5'-phosphosulfate synthase 2	Cytoplasm	enzyme	2.69E-05
-2.207	PEG10	paternally expressed 10	Nucleus	other	8.68E-08
2.007	PGF	placental growth factor	Extracellular Space	growth factor	6.26E-05
2.619	Prl2c2 (includes others)	prolactin family 2. subfamily c. member 2	Extracellular Space	growth factor	1.31E-04
2.387	Prl7b1	prolactin family 7. subfamily b. member 1	Extracellular Space	other	4.57E-03
-2.493	RBM3	RNA binding motif (RNP1. RRM) protein 3	Cytoplasm	other	2.44E-03
2.213	RNASE4	ribonuclease. RNase A family. 4	Extracellular Space	enzyme	8.27E-04
-3.041	Rnu3a	U3A small nuclear RNA	Other	other	6.03E-04
-2.355	RPL14	ribosomal protein L14	Cytoplasm	other	1.60E-03
-3.173	Rpph1	ribonuclease P RNA component H1	Other	other	2.42E-03
-2.063	Rprl2	ribonuclease P RNA-like 2	Other	other	2.99E-02
-2.297	Scarna13	small Cajal body-specific RNA 1	Other	other	1.28E-02
2.434	SCUBE2	signal peptide. CUB domain. EGF-like 2	Other	other	3.70E-06
2.008	SDC3	syndecan 3	Plasma Membrane	other	1.21E-03
2.045	Serpinb9f (includes others)	serine (or cysteine) peptidase inhibitor. clade B. member 9f	Cytoplasm	other	2.68E-03
2.576	Sh2d1b2	SH2 domain protein 1B2	Other	other	3.03E-03
2.016	SIKE1	suppressor of IKBKE 1	Cytoplasm	other	2.66E-06

2.054	SLC17A5	solute carrier family 17 (acidic sugar transporter). member 5	Plasma Membrane	transporter	1.71E-04
2.248	SLC25A12	solute carrier family 25 (aspartate/glutamate carrier). member 12	Cytoplasm	transporter	3.98E-04
3.411	SLC31A2	solute carrier family 31 (copper transporter). member 2	Plasma Membrane	transporter	2.31E-04
2.167	SLC38A3	solute carrier family 38. member 3	Plasma Membrane	transporter	6.52E-04
2.373	SLC38A6	solute carrier family 38. member 6	Other	transporter	1.30E-06
2.447	SLC44A5	solute carrier family 44. member 5	Plasma Membrane	transporter	3.20E-05
2.054	SLC6A4	solute carrier family 6 (neurotransmitter transporter). member 4	Plasma Membrane	transporter	6.65E-04
2.101	SLCO2A1	solute carrier organic anion transporter family. member 2A1	Plasma Membrane	transporter	9.10E-04
2.638	SLCO4C1	solute carrier organic anion transporter family. member 4C1	Plasma Membrane	transporter	1.69E-05
-2.915	Snora15	small nucleolar RNA. H/ACA box 15	Other	other	1.01E-02
-3.198	Snora17	small nucleolar RNA. H/ACA box 17	Other	other	8.75E-05
-2.433	Snora20	small nucleolar RNA. H/ACA box 20	Other	other	3.12E-03
-2.838	Snora21	small nucleolar RNA. H/ACA box 21	Other	other	1.77E-02
-2.487	Snora23	small nucleolar RNA. H/ACA box 23	Other	other	1.36E-02
-2.251	Snora28	small nucleolar RNA. H/ACA box 28	Other	other	3.80E-02
-2.651	Snora2b	small nucleolar RNA. H/ACA box 2B	Other	other	3.14E-03
-2.794	Snora30	small nucleolar RNA. H/ACA box 30	Other	other	4.33E-03
-3.247	Snora33	small nucleolar RNA. H/ACA box 33	Other	other	7.26E-03
-2.291	Snora34	small nucleolar RNA. H/ACA box 34	Other	other	1.82E-03
-4.888	Snora41	small nucleolar RNA. H/ACA box 41	Other	other	8.98E-06
-2.142	Snora43	small nucleolar RNA. H/ACA box 43	Other	other	4.41E-04
-2.313	Snora44	small nucleolar RNA. H/ACA box 44	Other	other	1.31E-02
-2.191	Snora5c	small nucleolar RNA. H/ACA box 5C	Other	other	7.29E-04
-2.046	Snora61	small nucleolar RNA. H/ACA box 61	Other	other	8.22E-04
-3.763	SNORA62	small nucleolar RNA. H/ACA box 62	Nucleus	other	5.66E-04
-2.605	Snora73a	small nucleolar RNA. H/ACA box 73a	Other	other	4.69E-03

-2.847	Snora73b	small nucleolar RNA. H/ACA box 73b	Other	other	4.33E-02
-3.708	SNORA74A	small nucleolar RNA. H/ACA box 74A	Nucleus	other	1.94E-04
-4.230	Snora75	small nucleolar RNA. H/ACA box 75	Other	other	3.62E-04
-4.689	Snora81	small nucleolar RNA. H/ACA box 81	Other	other	3.07E-03
-5.206	Snord118	small nucleolar RNA. C/D box 118	Other	other	1.13E-06
-2.508	Snord17	small nucleolar RNA. C/D box 17	Other	other	9.32E-06
-2.126	Snord2	small nucleolar RNA. C/D box 2	Other	other	1.24E-04
-2.266	Snord49a	small nucleolar RNA. C/D box 49A	Other	other	8.73E-05
-2.311	Snord65	small nucleolar RNA. C/D box 65	Other	other	8.18E-04
-2.373	Snord67	small nucleolar RNA. C/D box 67	Other	other	6.36E-04
2.282	Speer4a (includes others)	spermatogenesis associated glutamate (E)-rich protein 4a	Nucleus	other	1.77E-02
2.069	Srgn	serglycin	Cytoplasm	other	4.66E-03
-2.230	Tbrg3	transforming growth factor beta regulated gene 3	Other	other	5.85E-04
2.333	TC2N	tandem C2 domains. nuclear	Nucleus	transporter	3.48E-03
2.376	TNS4	tensin 4	Cytoplasm	other	2.57E-04
2.183	Tpsab1	tryptase alpha/beta 1	Nucleus	peptidase	7.82E-03
2.536	TSPAN18	tetraspanin 18	Other	other	2.67E-06
2.346	VPS33B	vacuolar protein sorting 33 homolog B (yeast)	Cytoplasm	transporter	3.66E-06
2.329	ZFAND2A	zinc finger. AN1-type domain 2A	Other	other	9.90E-06
-2.025	Zfp955a/Zfp955b	zinc finger protein 955B	Other	other	4.61E-02

Supplementary Table 2: Differentially expressed genes in E14.5 *Sprn*$^{0/0}$ placentas

Following normalization of the raw data, differentially expressed genes with a p-value <0.05 and a fold change (fc) inferior to -2 or superior to 2 were selected and are listed.

Fold Change	Symbol	Entrez Gene Name	Location	Type(s)	p-value
3.198	1300017J02Rik	RIKEN cDNA 1300017J02 gene	Extracellular Space	other	1.52E-06
2.839	Acnat1/Acnat2	acyl-coenzyme A amino acid N-acyltransferase 1	Cytoplasm	enzyme	3.17E-04
2.244	ACOX2	acyl-CoA oxidase 2. branched chain	Cytoplasm	enzyme	1.08E-06
2.042	ADH1C	alcohol dehydrogenase 1C (class I). gamma polypeptide	Cytoplasm	enzyme	4.02E-03
2.520	AGT	angiotensinogen (serpin peptidase inhibitor. clade A. member 8)	Extracellular Space	growth factor	7.46E-05
3.101	AHSG	alpha-2-HS-glycoprotein	Extracellular Space	other	1.66E-07
2.253	Akr1b7	aldo-keto reductase family 1. member B7	Cytoplasm	enzyme	1.87E-02
3.131	AKR1C3	aldo-keto reductase family 1. member C3	Cytoplasm	enzyme	4.52E-02
3.067	ALB	albumin	Extracellular Space	transporter	6.49E-07
3.344	ALDOB	aldolase B. fructose-bisphosphate	Cytoplasm	enzyme	1.47E-05
3.376	AMBP	alpha-1-microglobulin/bikunin precursor	Extracellular Space	transporter	2.10E-07
2.119	ANAPC13	anaphase promoting complex subunit 13	Nucleus	other	2.73E-02
2.470	APOA1	apolipoprotein A-I	Extracellular Space	transporter	1.22E-03
3.469	APOA2	apolipoprotein A-II	Extracellular Space	transporter	6.05E-07
2.933	APOA4	apolipoprotein A-IV	Extracellular Space	transporter	1.51E-04
2.072	APOB	apolipoprotein B	Extracellular Space	transporter	8.88E-04
2.531	Apoc1	apolipoprotein C-I	Extracellular Space	other	6.80E-05
3.091	APOC2	apolipoprotein C-II	Extracellular Space	transporter	3.10E-06
2.352	Apoc3	apolipoprotein C-III	Extracellular Space	transporter	4.56E-04
2.526	APOH	apolipoprotein H (beta-2-glycoprotein I)	Extracellular Space	transporter	1.40E-06
2.240	APOM	apolipoprotein M	Plasma Membrane	transporter	2.92E-04
2.160	Arxes1/Arxes2	adipocyte-related X-chromosome expressed sequence 2	Cytoplasm	other	3.08E-02
3.246	BEX1	brain expressed. X-linked 1	Other	other	4.26E-06
2.118	BEX4	brain expressed. X-linked 4	Cytoplasm	other	3.26E-02

2.279	BST1	bone marrow stromal cell antigen 1	Plasma Membrane	enzyme	2.19E-02
2.051	C10orf10	chromosome 10 open reading frame 10	Cytoplasm	other	1.76E-02
2.182	C14orf2	chromosome 14 open reading frame 2	Cytoplasm	other	9.88E-03
2.128	C6	complement component 6	Extracellular Space	other	4.71E-05
2.482	C9	complement component 9	Extracellular Space	other	2.28E-06
-2.065	CD300LD	CD300 molecule-like family member d	Plasma Membrane	other	5.94E-04
2.561	CDHR2	cadherin-related family member 2	Plasma Membrane	other	2.72E-06
-2.257	Ceacam15	carcinoembryonic antigen-related cell adhesion molecule 15	Other	other	2.10E-06
2.826	CFI	complement factor I	Extracellular Space	peptidase	1.42E-05
2.049	CIDEB	cell death-inducing DFFA-like effector b	Cytoplasm	other	4.12E-03
3.397	Clec2e/Clec2h	C-type lectin domain family 2. member h	Plasma Membrane	transmembrane receptor	4.52E-07
2.357	CMBL	carboxymethylenebutenolidase homolog (Pseudomonas)	Cytoplasm	enzyme	7.84E-04
2.024	COX20	COX20 cytochrome C oxidase assembly factor	Cytoplasm	other	2.77E-02
2.851	CPN1	carboxypeptidase N. polypeptide 1	Extracellular Space	peptidase	8.85E-07
2.401	CPS1	carbamoyl-phosphate synthase 1. mitochondrial	Cytoplasm	enzyme	8.76E-07
2.736	CUBN	cubilin (intrinsic factor-cobalamin receptor)	Plasma Membrane	transmembrane receptor	1.33E-06
2.425	CYP21A2	cytochrome P450. family 21. subfamily A. polypeptide 2	Cytoplasm	enzyme	1.82E-05
2.203	DAD1	defender against cell death 1	Cytoplasm	other	5.51E-03
2.600	DDX3Y	DEAD (Asp-Glu-Ala-Asp) box helicase 3. Y-linked	Other	enzyme	1.87E-02
5.100	Eif2s3y	eukaryotic translation initiation factor 2. subunit 3. structural gene Y-linked	Other	other	2.91E-04
2.072	EPS8L3	EPS8-like 3	Extracellular Space	other	1.87E-05
2.290	F2	coagulation factor II (thrombin)	Extracellular Space	peptidase	1.49E-06
2.086	Fam13a	family with sequence similarity 13. member A	Other	other	4.53E-02
2.948	FCGRT	Fc fragment of IgG. receptor. transporter. alpha	Plasma Membrane	transmembrane receptor	2.63E-04
3.588	FGA	fibrinogen alpha chain	Extracellular Space	other	7.99E-07
4.521	FGB	fibrinogen beta chain	Extracellular Space	other	4.83E-08
3.813	FGG	fibrinogen gamma chain	Extracellular Space	other	3.86E-08
2.174	Fxyd2	FXYD domain-containing ion transport regulator 2	Plasma Membrane	other	2.74E-04

5.231	GC	group-specific component (vitamin D binding protein)	Extracellular Space	transporter	2.39E-08
2.611	GIPC2	GIPC PDZ domain containing family. member 2	Cytoplasm	other	2.62E-06
2.977	GLP1R	glucagon-like peptide 1 receptor	Plasma Membrane	G-protein coupled receptor	3.69E-05
2.047	GSTK1	glutathione S-transferase kappa 1	Cytoplasm	enzyme	1.60E-02
3.622	GUCA2B	guanylate cyclase activator 2B (uroguanylin)	Extracellular Space	other	3.42E-02
3.004	HABP2	hyaluronan binding protein 2	Extracellular Space	peptidase	3.44E-06
5.834	Hamp/Hamp2	hepcidin antimicrobial peptide	Extracellular Space	other	5.73E-03
2.142	HBB	hemoglobin. beta	Cytoplasm	transporter	2.90E-03
2.412	HGD	homogentisate 1.2-dioxygenase	Cytoplasm	enzyme	1.25E-05
2.027	HRSP12	heat-responsive protein 12	Cytoplasm	other	1.92E-03
2.210	HSD3B2	hydroxy-delta-5-steroid dehydrogenase. 3 beta- and steroid delta-isomerase 2	Cytoplasm	enzyme	3.68E-09
2.625	ITIH2	inter-alpha-trypsin inhibitor heavy chain 2	Extracellular Space	other	3.46E-08
5.361	KLK3	kallikrein-related peptidase 3	Extracellular Space	peptidase	2.50E-02
3.455	KNG1	kininogen 1	Extracellular Space	other	2.92E-05
2.856	LRP2	low density lipoprotein receptor-related protein 2	Plasma Membrane	transporter	2.25E-07
2.592	LY96	lymphocyte antigen 96	Plasma Membrane	transmembrane receptor	3.85E-03
2.269	LYPD8	LY6/PLAUR domain containing 8	Other	other	2.23E-06
3.129	MAOB	monoamine oxidase B	Cytoplasm	enzyme	1.04E-05
2.249	NDUFA3	NADH dehydrogenase (ubiquinone) 1 alpha subcomplex. 3. 9kDa	Cytoplasm	enzyme	2.44E-02
2.290	NDUFB9	NADH dehydrogenase (ubiquinone) 1 beta subcomplex. 9. 22kDa	Cytoplasm	enzyme	3.90E-02
2.563	NDUFC1	NADH dehydrogenase (ubiquinone) 1. subcomplex unknown. 1. 6kDa	Cytoplasm	enzyme	1.41E-02
2.660	NDUFS6	NADH dehydrogenase (ubiquinone) Fe-S protein 6. 13kDa (NADH-coenzyme Q reductase)	Cytoplasm	enzyme	1.95E-02
2.007	NDUFS7	NADH dehydrogenase (ubiquinone) Fe-S protein 7. 20kDa (NADH-coenzyme Q reductase)	Cytoplasm	enzyme	4.88E-02
2.007	NNAT	neuronatin	Plasma Membrane	transporter	2.04E-02
3.289	OTC	ornithine carbamoyltransferase	Cytoplasm	enzyme	1.49E-09

3.460	PCBD1	pterin-4 alpha-carbinolamine dehydratase/dimerization cofactor of hepatocyte nuclear factor 1 alpha	Nucleus	transcription regulator	1.82E -04
-2.022	PCDHA2	protocadherin alpha 2	Plasma Membrane	other	3.74E -02
2.278	PDZK1IP1	PDZK1 interacting protein 1	Extracellula r Space	other	1.04E -03
2.038	PLA2G12B	phospholipase A2. group XIIB	Extracellula r Space	enzyme	3.08E -03
2.301	PLG	plasminogen	Extracellula r Space	peptidase	8.30E -06
2.546	PLGRKT	plasminogen receptor. C-terminal lysine transmembrane protein	Cytoplasm	other	1.31E -03
2.275	Prl5a1	prolactin family 5. subfamily a. member 1	Extracellula r Space	other	3.05E -03
3.903	PSCA	prostate stem cell antigen	Plasma Membrane	other	1.46E -03
-4.306	Psg18 (includes others)	pregnancy specific glycoprotein 18	Extracellula r Space	other	7.27E -11
3.759	RBP2	retinol binding protein 2. cellular	Cytoplasm	transporter	1.13E -07
2.084	RBP4	retinol binding protein 4. plasma	Extracellula r Space	transporter	1.72E -05
-2.485	Rnu3a	U3A small nuclear RNA	Other	other	3.86E -03
-2.374	Rnu73b	U73B small nuclear RNA	Other	other	2.85E -02
2.229	Rpl29 (includes others)	ribosomal protein L29	Cytoplasm	other	2.55E -02
2.248	RRAS	related RAS viral (r-ras) oncogene homolog	Cytoplasm	enzyme	9.05E -03
-2.811	Scarna13	small Cajal body-specific RNA 1	Other	other	2.53E -03
-2.074	Scarna2	small Cajal body-specific RNA 2	Other	other	1.44E -02
-3.363	Scarna6	small Cajal body-specific RNA 6	Other	other	2.59E -05
3.780	SCGB1A1	secretoglobin. family 1A. member 1 (uteroglobin)	Extracellula r Space	cytokine	2.42E -03
6.231	SERPINA1	serpin peptidase inhibitor. clade A (alpha-1 antiproteinase. antitrypsin). member 1	Extracellula r Space	other	1.12E -06
2.085	SERPINA10	serpin peptidase inhibitor. clade A (alpha-1 antiproteinase. antitrypsin). member 10	Extracellula r Space	other	2.84E -02
4.455	SERPIND1	serpin peptidase inhibitor. clade D (heparin cofactor). member 1	Extracellula r Space	other	7.45E -11
2.551	SLC22A2	solute carrier family 22 (organic cation transporter). member 2	Plasma Membrane	transporter	1.63E -05
2.951	SLC27A2	solute carrier family 27 (fatty acid transporter). member 2	Cytoplasm	transporter	2.23E -03

2.278	SLC2A2	solute carrier family 2 (facilitated glucose transporter). member 2	Plasma Membrane	transporter	8.31E-06
2.145	SLC39A5	solute carrier family 39 (zinc transporter). member 5	Plasma Membrane	transporter	8.86E-04
2.590	SLC3A1	solute carrier family 3 (amino acid transporter heavy chain). member 1	Plasma Membrane	transporter	3.81E-07
2.102	SLC5A1	solute carrier family 5 (sodium/glucose cotransporter). member 1	Plasma Membrane	transporter	1.24E-03
2.680	SLC7A9	solute carrier family 7 (amino acid transporter light chain. bo.+ system). member 9	Plasma Membrane	transporter	7.61E-08
2.272	SLCO1B3	solute carrier organic anion transporter family. member 1B3	Plasma Membrane	transporter	7.01E-07
2.782	SLCO4C1	solute carrier organic anion transporter family. member 4C1	Plasma Membrane	transporter	7.58E-06
2.414	SMS	spermine synthase	Cytoplasm	enzyme	1.28E-03
-2.294	Snora16a	small nucleolar RNA. H/ACA box 16A	Other	other	3.47E-04
-3.214	Snora73a	small nucleolar RNA. H/ACA box 73a	Other	other	7.91E-04
-4.167	Snora73b	small nucleolar RNA. H/ACA box 73b	Other	other	7.20E-03
-2.087	Snord13	small nucleolar RNA. C/D box 13	Other	other	3.08E-02
-4.937	Snord45b	small nucleolar RNA. C/D box 45B	Other	other	7.53E-03
-2.371	Snord73a	small nucleolar RNA. C/D box U73A	Other	other	5.05E-03
-2.189	Snord99	small nucleolar RNA. C/D box 99	Other	other	1.70E-02
3.833	SPINK1	serine peptidase inhibitor. Kazal type 1	Extracellular Space	other	5.79E-06
3.743	SPP2	secreted phosphoprotein 2. 24kDa	Extracellular Space	other	2.37E-06
2.203	STRA13	stimulated by retinoic acid 13	Nucleus	other	4.25E-03
2.028	SUSD2	sushi domain containing 2	Extracellular Space	other	5.75E-03
2.272	Tceb2	transcription elongation factor B (SIII). polypeptide 2	Nucleus	transcription regulator	3.95E-02
2.050	TF	transferrin	Extracellular Space	transporter	2.66E-04
2.248	TINAG	tubulointerstitial nephritis antigen	Extracellular Space	peptidase	2.89E-09
2.894	TMA7	translation machinery associated 7 homolog (S. cerevisiae)	Other	other	1.72E-02
2.330	Tma7-ps	translational machinery associated 7 homolog (S. cerevisiae). pseudogene	Other	other	4.29E-02
-2.138	Tmem181c-ps	transmembrane protein 181C. pseudogene	Other	other	3.66E-02

4.121	TTR	transthyretin	Extracellular Space	transporter	9.75E-07
3.192	UGT2B10	UDP glucuronosyltransferase 2 family. polypeptide B10	Cytoplasm	enzyme	1.73E-09
3.088	UGT3A1	UDP glycosyltransferase 3 family. polypeptide A1	Other	enzyme	2.03E-10
3.202	Uty	ubiquitously transcribed tetratricopeptide repeat gene. Y chromosome	Nucleus	other	2.12E-03
2.305	VDR	vitamin D (1.25-dihydroxyvitamin D3) receptor	Nucleus	transcription regulator	4.47E-04
2.988	VTN	vitronectin	Extracellular Space	other	3.07E-05
2.025	ZNRD1	zinc ribbon domain containing 1	Nucleus	transcription regulator	2.54E-02

Supplementary Table 3 : Placenta RT-qPCR confirmation of microarray results

RT-qPCR was performed on 2 differentially expressed genes and 3 non differentially expressed genes according to microarray analysis, using 3 individual placentas for each genotypes (see Material and Methods). *: t test p<0.05

**Gene relative arbitrary expression levels / Genotype of Placenta**	**WT (mean +/- SD)**	***Sprn*$^{0/0}$ (mean +/- SD)**
TBPA	154.19 +/- 36.04	170.08 +/- 37.11
Gli3	75.16 +/- 13.77	71.9 +/- 18.98
GC	72.20 +/- 49.74	284.96 +/- 212.55*
PSG23	192.81 +/- 36.35	174.58 +/- 75.03
SerpinA1	17.95 +/- 17.91	57.09 +/- 49.05*

Supplementary Table 4 : Milk protein concentrations.

Milk protein concentration was estimated on 3 milk samples collected at L7.5 from 3 different mice of each genotype (see Material and Method section)

Milk Sample	Protein concentration µg/µl
FVB/NJ	54.8
FVB/NJ	53.0
FVB/NJ	67.1
$\mathit{Sprn}^{0/0}$	44.4
$\mathit{Sprn}^{0/0}$	47.9
$\mathit{Sprn}^{0/0}$	64.3

Supplementary Table 5: Differentially expressed genes in G7.5 *Sprn$^{0/0}$* mammary glands

Following normalization of the raw data, differentially expressed genes with a p-value <0.05 and a fold change (fc) inferior to -2 or superior to 2 were selected and are listed.

Fold Change	ID	Symbol	Entrez Gene Name	Location	Type(s)
2.421	69206	2010016I18Rik	RIKEN cDNA 2010016I18 gene	Other	other
-3.603	100302567	AF357399	snoRNA AF357399	Other	other
-2.007	71898	Apol9a/Apol9b	apolipoprotein L 9b	Other	other
-2.839	12228	BTG3	BTG family, member 3	Nucleus	other
2.215	69165	Cd209b	CD209b antigen	Plasma Membrane	other
2.893	12683	CIDEA	cell death-inducing DFFA-like effector a	Cytoplasm	other
2.104	12865	COX7A1	cytochrome c oxidase subunit VIIa polypeptide 1 (muscle)	Cytoplasm	enzyme
2.175	12686	ELOVL3	ELOVL fatty acid elongase 3	Cytoplasm	enzyme
2.321	14069	F8	coagulation factor VIII, procoagulant component	Extracellular Space	peptidase
-2.327	328825	Gm5093	predicted gene 5093	Other	other
-2.257	666634	Gm8203	predicted pseudogene 8203	Other	other
-3.403	15039	H2-T22	histocompatibility 2, T region locus 22	Other	other
-2.773	51788	H2AFZ	H2A histone family, member Z	Nucleus	other
-2.027	319189	HIST2H2BF	histone cluster 2, H2bf	Nucleus	other
-3.374	100504404	HLA-DRA	major histocompatibility complex, class II, DR alpha	Plasma Membrane	transmembrane receptor
-2.263	15040	HLA-E	major histocompatibility complex, class I, E	Plasma Membrane	transmembrane receptor
-2.222	236312	IFI16	interferon, gamma-inducible protein 16	Nucleus	transcription regulator
4.152	16017	IGHG1	immunoglobulin heavy constant gamma 1 (G1m marker)	Extracellular Space	other

9.725	16016	Ighg2b	immunoglobulin heavy constant gamma 2B	Extracellular Space	other
4.937	380795	Ighg3	Immunoglobulin heavy constant gamma 3	Other	other
2.460	777686	Ighv2-2	immunoglobulin heavy variable 2-2	Other	other
6.420	16069	IGJ	immunoglobulin J polypeptide, linker protein for immunoglobulin alpha and mu polypeptides	Extracellular Space	other
3.167	16071	IGKC	immunoglobulin kappa constant	Extracellular Space	other
4.309	110763	Igkj5	immunoglobulin kappa joining 5	Other	other
8.484	381777	Igkv1-110	immunoglobulin kappa variable 1-110	Other	other
14.837	16098	Igkv1-117	immunoglobulin kappa variable 1-117	Other	other
3.790	243420	Igkv1-135	immunoglobulin kappa variable 1-135	Other	other
4.769	384515	Igkv4-68	immunoglobulin kappa variable 4-68	Plasma Membrane	other
6.729	667881	Igkv6-14	immunoglobulin kappa variable 6-14	Other	other
5.697	16142	Iglv1	immunoglobulin lambda variable 1	Other	other
6.969	110612	Iglv2	immunoglobulin lambda variable 2	Other	other
2.197	387163	mir-145	microRNA 145	Cytoplasm	microRNA
-2.255	66128	MRPS36	mitochondrial ribosomal protein S36	Cytoplasm	other
2.692	60361	Ms4a4b (includes others)	membrane-spanning 4-domains, subfamily A, member 4B	Plasma Membrane	other
2.781	93714	PCDHGA6	protocadherin gamma subfamily A, 6	Extracellular Space	other
2.052	237928	PHOSPHO1	phosphatase, orphan 1	Extracellular Space	enzyme
2.507	19017	PPARGC1A	peroxisome proliferator-activated receptor gamma, coactivator 1 alpha	Nucleus	transcription regulator

2.007	245195	Retnlg	resistin like gamma	Extracellular Space	other
-2.713	19850	Rnu3a	U3A small nuclear RNA	Other	other
2.334	26458	SLC27A2	solute carrier family 27 (fatty acid transporter), member 2	Cytoplasm	transporter
2.625	56485	SLC2A5	solute carrier family 2 (facilitated glucose/fructose transporter), member 5	Plasma Membrane	transporter
2.234	54403	SLC4A4	solute carrier family 4 (sodium bicarbonate cotransporter), member 4	Plasma Membrane	transporter
-2.291	100379145	Snora23	small nucleolar RNA, H/ACA box 23	Other	other
-2.934	104368	SNORA70	small nucleolar RNA, H/ACA box 70	Other	other
-2.156	100306944	Snora73a	small nucleolar RNA, H/ACA box 73a	Other	other
-2.161	436583	SNORA74A	small nucleolar RNA, H/ACA box 74A	Nucleus	other
-2.970	100217420	Snora81	small nucleolar RNA, H/ACA box 81	Other	other
-2.083	100217422	Snord13	small nucleolar RNA, C/D box 13	Other	other
-3.515	100217468	Snord45b	small nucleolar RNA, C/D box 45B	Other	other
-2.371	100217462	Snord92	small nucleolar RNA, C/D box 92	Other	other
-2.100	21990	TPH1	tryptophan hydroxylase 1	Nucleus	enzyme
7.158	22227	UCP1	uncoupling protein 1 (mitochondrial, proton carrier)	Cytoplasm	transporter
-2.064	230590	ZYG11A	zyg-11 family member A, cell cycle regulator	Other	other
